# Graph Theoretical Analysis Reveals: Women’s Brains Are Better Connected than Men’s

**DOI:** 10.1371/journal.pone.0130045

**Published:** 2015-07-01

**Authors:** Balázs Szalkai, Bálint Varga, Vince Grolmusz

**Affiliations:** 1 PIT Bioinformatics Group, Eötvös University, H-1117 Budapest, Hungary; 2 Uratim Ltd., H-1118 Budapest, Hungary; Nankai University, CHINA

## Abstract

Deep graph-theoretic ideas in the context with the graph of the World Wide Web led to the definition of Google’s PageRank and the subsequent rise of the most popular search engine to date. Brain graphs, or connectomes, are being widely explored today. We believe that non-trivial graph theoretic concepts, similarly as it happened in the case of the World Wide Web, will lead to discoveries enlightening the structural and also the functional details of the animal and human brains. When scientists examine large networks of tens or hundreds of millions of vertices, only fast algorithms can be applied because of the size constraints. In the case of diffusion MRI-based structural human brain imaging, the effective vertex number of the connectomes, or brain graphs derived from the data is on the scale of several hundred today. That size facilitates applying strict mathematical graph algorithms even for some hard-to-compute (or NP-hard) quantities like vertex cover or balanced minimum cut. In the present work we have examined brain graphs, computed from the data of the Human Connectome Project, recorded from male and female subjects between ages 22 and 35. Significant differences were found between the male and female structural brain graphs: we show that the average female connectome has more edges, is a better expander graph, has larger minimal bisection width, and has more spanning trees than the average male connectome. Since the average female brain weighs less than the brain of males, these properties show that the female brain has better graph theoretical properties, in a sense, than the brain of males. It is known that the female brain has a smaller gray matter/white matter ratio than males, that is, a larger white matter/gray matter ratio than the brain of males; this observation is in line with our findings concerning the number of edges, since the white matter consists of myelinated axons, which, in turn, roughly correspond to the connections in the brain graph. We have also found that the minimum bisection width, normalized with the edge number, is also significantly larger in the right and the left hemispheres in females: therefore, the differing bisection widths are independent from the difference in the number of edges.

## Introduction

In the last several years hundreds of publications appeared describing or analyzing structural or functional networks of the brain, frequently referred to as “connectome” [1–4]. Some of these publications analyzed data from healthy humans [5–8], and some compared the connectome of the healthy brain with diseased one [9–13].

So far, the analyses of the connectomes mostly used tools developed for very large networks, such as the graph of the World Wide Web (with billions of vertices), or protein-protein interaction networks (with tens or hundreds of thousands of vertices), and because of the huge size of original networks, these methods used only very fast algorithms and frequently just primary degree statistics and graph-edge counting between pre-defined regions or lobes of the brain [14].

In the present work we demonstrate that deep and more intricate graph theoretic parameters could also be computed by using, among other tools, contemporary integer programming approaches for connectomes with several hundred vertices.

With these mathematical tools we show statistically significant differences in some graph properties of the connectomes, computed from MRI imaging data of male and female brains. We will not try to associate behavioral patterns of males and females with the discovered structural differences [14] (see also the debate that article has generated: [15–17]), because we do not have behavioral data of the subjects of the imaging study, and, additionally, we cannot describe high-level functional properties implied by those structural differences. However, we clearly demonstrate that deep graph-theoretic parameters show “better” connections in a certain sense in female connectomes than in male ones.

The study of [14] analyzed the 95-vertex graphs of 949 subjects aged between 8 and 22 years, using basic statistics for the numbers of edges running either between or within different lobes of the brain (the parameters deduced were called *hemispheric connectivity ratio, modularity, transitivity and participation coefficients*, see [14] for the definitions). It was found that males have significantly more intra-hemispheric edges than females, while females have significantly more inter-hemispheric edges than males.

## Results and Discussion

We have analyzed the connectomes of 96 subjects, 52 females and 44 males, each with 83, 129, 234, 463 and 1015 node resolutions, and each graphs with five different weight functions. We considered the connectomes as graphs with weighted edges, and performed graph-theoretic analyses with computing some polynomial-time computable and also some NP-hard graph parameters on the individual graphs, and then compared the results statistically for the male and the female group.

We have found that female connectomes have more edges, larger (edge-normalized) minimum bisection widths, larger minimum-vertex covers and more spanning trees and are better expanders than the male connectomes.

In order to describe the parameters, which differ significantly among male and female connectomes, we need to place them in the context of their graph theoretical definitions.

### Edge number and edge weights

We have found significantly higher number of edges (counted with 5 types of weights and also without any weights) in both hemispheres and also in the whole brain in females, in all resolutions. This finding is surprising, since we used the same parcellation and the same tractography and the same graph-construction methods for female and male brains, and because it is proven that females have, on average, less-weighting brains than males [18]. For example, in the 234-vertex resolution, the average number of (unweighted) edges in female connectomes is 1826, in males 1742, with *p* = 0.00063 (see Table 1 with a summary and Tables 2, 3, 4, 5 and 6 with the results). The work of [14] reported similar findings in inter-hemispheric connections only.

**Table 1:** The results and the statistical analysis of the graph-theoretical evaluation of the sex differences in the 96 diffusion MRI images. The first column gives the resolutions in each hemisphere; the numbers of nodes in the whole graph are 83, 129, 234, 463 and 1015. The second column describes the graph parameter computed: its syntactics is as follows: each parameter-name contains two separating “_” symbols that define three parts of the parameter-name. The first part describe the hemisphere or the whole connectome with the words Left, Right or All. The second part describes the parameter computed, and the third part the weight function used (their definitions are given in section “Materials and methods”). The third column contains the p-values of the first round, the second column the p-values of the second round, and the third column the (very strict) Holm-Bonferroni correction of the p-value. With p = 0.05 *all* the first 12 rows describe significantly different graph theoretical properties between sexes. One-by-one, each row with italic third column describe significant differences between sexes, with p = 0.05. For the details we refer to the section “Statistical analysis”.

Scale	Property	p (1st)	p (2nd)	p (corrected)
129	Right_MinCutBalDivSum_FAMean	0.00807	*0.00003*	**0.00493**
89	All_LogSpanningForestN_FiberNDivLength	0.00003	*0.00004*	**0.00555**
234	All_PGEigengap_FiberNDivLength	0.00321	*0.00007*	**0.00984**
129	All_PGEigengap_FiberNDivLength	0.00792	*0.00011*	**0.01610**
89	Left_MinCutBalDivSum_FiberN	0.00403	*0.00011*	**0.01608**
89	Right_MinCutBalDivSum_FAMean	0.00496	*0.00015*	**0.02161**
129	Left_PGEigengap_FiberNDivLength	0.00223	*0.00015*	**0.02231**
234	All_PGEigengap_FiberN	0.00826	*0.00022*	**0.03130**
89	All_Sum_Unweighted	0.00025	*0.00022*	**0.03119**
129	Left_MinCutBalDivSum_FiberN	0.00001	*0.00023*	**0.03198**
89	All_LogSpanningForestN_FiberN	0.00001	*0.00028*	**0.03855**
89	Right_Sum_FAMean	0.00028	*0.00029*	**0.04037**
234	All_Sum_Unweighted	0.00063	*0.00032*	**0.04406**
234	Left_PGEigengap_FiberNDivLength	0.00013	*0.00038*	0.05243
129	All_Sum_Unweighted	0.00026	*0.00042*	0.05746
234	All_Sum_FAMean	0.00014	*0.00047*	0.06293
129	All_LogSpanningForestN_FiberN	0.00000	*0.00048*	0.06377
89	All_Sum_FAMean	0.00029	*0.00050*	0.06663
129	Right_Sum_FAMean	0.00062	*0.00051*	0.06796
234	Right_PGEigengap_FiberNDivLength	0.00041	*0.00053*	0.06886
89	Left_Sum_Unweighted	0.00378	*0.00068*	0.08840
234	Right_Sum_FAMean	0.00085	*0.00084*	0.10797
234	Left_Sum_Unweighted	0.00293	*0.00092*	0.11791
129	All_Sum_FAMean	0.00015	*0.00097*	0.12380
234	Left_MinCutBalDivSum_FiberN	0.00002	*0.00108*	0.13550
89	Left_LogSpanningForestN_FiberNDivLength	0.00343	*0.00116*	0.14528
89	All_LogSpanningForestN_Unweighted	0.00113	*0.00121*	0.15021
234	Left_MinCutBalDivSum_FiberLengthMean	0.00411	*0.00123*	0.15078
89	All_LogSpanningForestN_FAMean	0.00012	*0.00126*	0.15345
463	Left_MinCutBalDivSum_FiberN	0.00062	*0.00127*	0.15316
89	Right_Sum_Unweighted	0.00019	*0.00128*	0.15344
129	Left_MinCutBalDivSum_Unweighted	0.00265	*0.00134*	0.15975
463	Left_MinCutBalDivSum_FiberLengthMean	0.00655	*0.00135*	0.15922
89	Left_MinCutBalDivSum_Unweighted	0.00206	*0.00136*	0.15905
129	Left_PGEigengap_FiberN	0.00382	*0.00142*	0.16465
463	All_Sum_FAMean	0.00297	*0.00147*	0.16947
234	All_LogSpanningForestN_FAMean	0.00043	*0.00150*	0.17091
234	Left_PGEigengap_FiberN	0.00066	*0.00163*	0.18451
129	Right_LogSpanningForestN_FAMean	0.00143	*0.00170*	0.19013
89	Left_MinCutBalDivSum_FiberNDivLength	0.00031	*0.00175*	0.19390
129	All_LogSpanningForestN_FiberNDivLength	0.00000	*0.00177*	0.19424
129	All_LogSpanningForestN_Unweighted	0.00218	*0.00182*	0.19827
129	Right_Sum_Unweighted	0.00068	*0.00186*	0.20060
129	Left_PGEigengap_FAMean	0.00995	*0.00191*	0.20478
129	All_LogSpanningForestN_FAMean	0.00019	*0.00211*	0.22369
234	Left_Sum_FAMean	0.00026	*0.00212*	0.22284
89	Right_LogSpanningForestN_FAMean	0.00067	*0.00239*	0.24805
234	Left_PGEigengap_FAMean	0.00141	*0.00240*	0.24672
89	Left_PGEigengap_Unweighted	0.00458	*0.00243*	0.24822
129	Left_MinCutBalDivSum_FiberLengthMean	0.00892	*0.00245*	0.24713
463	Left_MinCutBalDivSum_Unweighted	0.00153	*0.00259*	0.25859
89	Left_Sum_FAMean	0.00056	*0.00279*	0.27579
234	Left_MinCutBalDivSum_Unweighted	0.00154	*0.00289*	0.28281
234	Left_PGEigengap_FiberLengthMean	0.00554	*0.00295*	0.28590
234	Right_LogSpanningForestN_FAMean	0.00380	*0.00305*	0.29247
234	Left_PGEigengap_Unweighted	0.00176	*0.00338*	0.32152
89	Left_PGEigengap_FAMean	0.00215	*0.00359*	0.33776
89	Left_LogSpanningForestN_FiberN	0.00012	*0.00395*	0.36754
1015	Left_MinCutBalDivSum_Unweighted	0.00844	*0.00395*	0.36377
129	Left_Sum_Unweighted	0.00232	*0.00456*	0.41494
89	Left_LogSpanningForestN_FAMean	0.00082	*0.00496*	0.44613
234	Right_MinCutBalDivSum_Unweighted	0.00462	*0.00543*	0.48309
463	All_MinSpanningForest_FAMean	0.00151	*0.00576*	0.50669
89	Right_LogSpanningForestN_FiberNDivLength	0.00022	*0.00587*	0.51103
234	Left_LogSpanningForestN_FAMean	0.0006	*0.00595*	0.51135
463	Right_MinSpanningForest_FAMean	0.00435	*0.00607*	0.51554
234	Right_PGEigengap_Unweighted	0.00095	*0.00626*	0.52613
129	Left_Sum_FAMean	0.00032	*0.00660*	0.54763
89	Left_AdjLMaxDivD_FiberN	0.00501	*0.00804*	0.65922
234	Right_Sum_Unweighted	0.00224	*0.00845*	0.68434
234	Right_PGEigengap_FiberN	0.00009	*0.00910*	0.72774
129	All_Sum_FiberN	0.00000	*0.00938*	0.74121
234	Right_PGEigengap_FAMean	0.00074	*0.00974*	0.76000
129	Right_PGEigengap_FAMean	0.00296	*0.00981*	0.75533
89	Right_PGEigengap_Unweighted	0.00087	*0.01053*	0.79992
129	Right_MinCutBalDivSum_FiberN	0.00563	*0.01101*	0.82545
129	Right_MinCutBalDivSum_Unweighted	0.00492	*0.01212*	0.89675
129	Left_LogSpanningForestN_FAMean	0.00106	*0.01218*	0.88946
129	Left_LogSpanningForestN_FiberN	0.00014	*0.01258*	0.90543
89	All_Sum_FiberN	0.00000	*0.01290*	0.91561
234	All_Sum_FiberN	0.00000	*0.01358*	0.95029
1015	Left_MinCutBalDivSum_FiberN	0.00320	*0.01379*	0.95167
463	Right_Sum_FAMean	0.00745	*0.01407*	0.95680
89	Right_LogSpanningForestN_Unweighted	0.00541	*0.01438*	0.96326
129	Left_LogSpanningForestN_FiberNDivLength	0.00288	*0.01447*	0.95482
129	Right_PGEigengap_Unweighted	0.00242	*0.01676*	1.08923
129	Right_PGEigengap_FiberN	0.00869	*0.01706*	1.09156
1015	Right_HoffmanBound_FiberN	0.00046	*0.01707*	1.07534
234	All_MinVertexCover_FAMean	0.00289	*0.01713*	1.06207
463	Right_HoffmanBound_FiberN	0.00150	*0.01941*	1.18391
89	All_HoffmanBound_FAMean	0.00087	*0.02011*	1.20644
89	All_Sum_FiberNDivLength	0.00002	*0.02117*	1.24924
463	All_Sum_FiberN	0.00000	*0.02195*	1.27314
234	Right_MinCutBalDivSum_FiberN	0.00234	*0.02197*	1.25212
89	Right_LogSpanningForestN_FiberN	0.00083	*0.02539*	1.42194
234	Right_MinCutBalDivSum_FiberLengthMean	0.00234	*0.02663*	1.46442
89	Right_MinCutBalDivSum_FiberNDivLength	0.00072	*0.02854*	1.54108
1015	All_Sum_FiberN	0.00000	*0.02893*	1.53335
129	Left_MinCutBalDivSum_FiberNDivLength	0.00019	*0.02897*	1.50652
89	Right_PGEigengap_FAMean	0.00112	*0.02948*	1.50336
1015	All_LogSpanningForestN_FiberNDivLength	0.00224	*0.03016*	1.50823
234	All_LogSpanningForestN_FiberN	0.00091	*0.03308*	1.62113
234	Right_PGEigengap_FiberLengthMean	0.00367	*0.03369*	1.61706
129	Right_MinCutBalDivSum_FiberLengthMean	0.00768	*0.04500*	2.11516
129	All_Sum_FiberNDivLength	0.00008	*0.04728*	2.17484
129	Right_LogSpanningForestN_FiberNDivLength	0.00051	*0.04891*	2.20103
234	All_LogSpanningForestN_FiberNDivLength	0.00106	0.05095	2.24168
129	Right_LogSpanningForestN_FiberN	0.00045	0.05578	2.39838
1015	Left_LogSpanningForestN_FiberNDivLength	0.00208	0.05932	2.49129
89	Right_MinCutBalDivSum_FiberN	0.00346	0.06284	2.57642
89	Right_HoffmanBound_FiberNDivLength	0.0056	0.06309	2.52357
89	Right_PGEigengap_FiberLengthMean	0.00949	0.06515	2.54092
463	Left_MinSpanningForest_FAMean	0.00239	0.06537	2.48399
234	Left_MinCutBalDivSum_FiberNDivLength	0.00642	0.06548	2.42270
1015	Right_HoffmanBound_FiberNDivLength	0.00443	0.06730	2.42270
234	Left_MinVertexCover_FAMean	0.00107	0.07139	2.49865
234	All_Sum_FiberNDivLength	0.00044	0.07318	2.48798
89	Right_Sum_FiberN	0.00000	0.07799	2.57379
89	Right_Sum_FiberNDivLength	0.00018	0.07920	2.53454
129	Left_Sum_FiberN	0.00000	0.08380	2.59777
129	Right_Sum_FiberN	0.00001	0.08653	2.59588
129	Left_HoffmanBound_Unweighted	0.00848	0.08944	2.59364
89	Left_Sum_FiberN	0.00000	0.09430	2.64039
234	Left_Sum_FiberN	0.00040	0.11447	3.09078
129	Right_Sum_FiberNDivLength	0.00180	0.12102	3.14639
463	All_Sum_FiberNDivLength	0.00139	0.15116	3.77901
1015	Right_MinCutBalDivSum_FiberN	0.00046	0.16276	3.90634
234	Right_Sum_FiberN	0.00012	0.16411	3.77450
89	Left_Sum_FiberNDivLength	0.00043	0.16774	3.69028
463	Left_Sum_FiberN	0.00107	0.16844	3.53733
1015	Left_Sum_FiberN	0.00199	0.18957	3.79141
463	Right_Sum_FiberN	0.00050	0.20907	3.97234
463	Right_MinCutBalDivSum_FiberN	0.00641	0.21629	3.89328
129	Left_Sum_FiberNDivLength	0.00100	0.22542	3.83211
1015	All_MinVertexCover_FiberN	0.00196	0.22749	3.63992
1015	All_Sum_FiberNDivLength	0.00311	0.23379	3.50685
234	Right_Sum_FiberNDivLength	0.00562	0.23691	3.31678
1015	Right_Sum_FiberN	0.00073	0.28752	3.73781
89	Right_HoffmanBound_FAMean	0.00587	0.32069	3.84830
89	All_MinVertexCoverBinary_Unweighted	0.00716	0.38829	4.27116
234	Right_LogSpanningForestN_FiberNDivLength	0.00940	0.40996	4.09964
89	Left_HoffmanBound_FiberN	0.00175	0.41913	3.77221
89	All_MinVertexCover_FiberNDivLength	0.00036	0.46677	3.73420
89	Right_MinSpanningForest_FiberLengthMean	0.00491	0.55239	3.86672
234	Right_MinSpanningForest_FiberLengthMean	0.00601	0.55631	3.33785
463	All_MinVertexCover_FiberN	0.00056	0.60428	3.02138
129	All_MinVertexCover_FiberN	0.00232	0.71406	2.85623
89	All_MinVertexCover_FiberN	0.00244	0.84437	1.68874
234	All_MinVertexCover_FiberN	0.00055	0.92958	0.92958

**Table 2:** The graph-theoretic parameters computed for the 83-vertex graphs. The table contains their arithmetic means in the male and female groups, and the corresponding p-values in round 1 (see the “Statistical analysis” subsection). The results of the graph-parameters are defined in the caption of Table 1. Significant differences (*p* < 0.01) are denoted with an asterisk in the last column.

Property	Female	Male	p-value
All_AdjLMaxDivD_FAMean	1.36008	1.37750	0.06806
All_AdjLMaxDivD_FiberLengthMean	1.44214	1.43602	0.72030
All_AdjLMaxDivD_FiberN	2.02416	2.10529	0.05606
All_AdjLMaxDivD_FiberNDivLength	1.84476	1.86864	0.41834
All_AdjLMaxDivD_Unweighted	1.26760	1.26456	0.63251
All_HoffmanBound_FAMean	4.36096	4.18564	0.00087 *
All_HoffmanBound_FiberLengthMean	3.21938	3.26552	0.33136
All_HoffmanBound_FiberN	2.63525	2.55573	0.03144
All_HoffmanBound_FiberNDivLength	2.51038	2.40550	0.01815
All_HoffmanBound_Unweighted	4.55192	4.43931	0.04616
All_LogSpanningForestN_FAMean	110.69890	101.82758	0.00012 *
All_LogSpanningForestN_FiberLengthMean	456.60084	452.95875	0.18687
All_LogSpanningForestN_FiberN	397.53780	389.79037	0.00001 *
All_LogSpanningForestN_FiberNDivLength	148.03174	139.85355	0.00003 *
All_LogSpanningForestN_Unweighted	191.66035	187.85180	0.00113 *
All_MinCutBalDivSum_FAMean	0.00793	0.00474	0.14869
All_MinCutBalDivSum_FiberLengthMean	0.03115	0.02889	0.47008
All_MinCutBalDivSum_FiberN	0.02924	0.02711	0.34092
All_MinCutBalDivSum_FiberNDivLength	0.02868	0.02644	0.38768
All_MinCutBalDivSum_Unweighted	0.04001	0.03721	0.28887
All_MinSpanningForest_FAMean	19.78188	18.63722	0.02232
All_MinSpanningForest_FiberLengthMean	1096.37958	1112.97289	0.10506
All_MinSpanningForest_FiberN	99.53846	102.93333	0.14280
All_MinSpanningForest_FiberNDivLength	3.65548	3.66822	0.93669
All_MinVertexCoverBinary_Unweighted	59.80769	59.00000	0.00716 *
All_MinVertexCover_FAMean	18.73144	18.10619	0.01699
All_MinVertexCover_FiberLengthMean	2014.06431	1955.70824	0.37460
All_MinVertexCover_FiberN	2427.21154	2315.20000	0.00244 *
All_MinVertexCover_FiberNDivLength	110.25657	103.59777	0.00036 *
All_MinVertexCover_Unweighted	40.90385	41.00000	0.32897
All_PGEigengap_FAMean	0.05403	0.05071	0.28914
All_PGEigengap_FiberLengthMean	0.04167	0.03891	0.43309
All_PGEigengap_FiberN	0.03156	0.02829	0.03885
All_PGEigengap_FiberNDivLength	0.03470	0.03062	0.01847
All_PGEigengap_Unweighted	0.05214	0.04740	0.09708
All_Sum_FAMean	222.01291	201.02562	0.00029 *
All_Sum_FiberLengthMean	16845.33062	15792.24352	0.06219
All_Sum_FiberN	11261.65385	10237.13333	0.00000 *
All_Sum_FiberNDivLength	476.56342	433.37987	0.00002 *
All_Sum_Unweighted	567.07692	539.80000	0.00025 *
Left_AdjLMaxDivD_FAMean	1.33644	1.35216	0.15767
Left_AdjLMaxDivD_FiberLengthMean	1.40515	1.38890	0.32795
Left_AdjLMaxDivD_FiberN	1.90607	2.02087	0.00501 *
Left_AdjLMaxDivD_FiberNDivLength	1.71498	1.77482	0.07539
Left_AdjLMaxDivD_Unweighted	1.24027	1.23523	0.43598
Left_HoffmanBound_FAMean	4.55406	4.38621	0.01297
Left_HoffmanBound_FiberLengthMean	3.25098	3.28435	0.51250
Left_HoffmanBound_FiberN	2.71430	2.61098	0.00175 *
Left_HoffmanBound_FiberNDivLength	2.66652	2.59451	0.13782
Left_HoffmanBound_Unweighted	4.73205	4.57434	0.01379
Left_LogSpanningForestN_FAMean	53.30579	48.82905	0.00082 *
Left_LogSpanningForestN_FiberLengthMean	229.63370	227.32675	0.18765
Left_LogSpanningForestN_FiberN	199.27958	195.25428	0.00012 *
Left_LogSpanningForestN_FiberNDivLength	73.53683	69.82889	0.00343 *
Left_LogSpanningForestN_Unweighted	95.46307	93.39767	0.01389
Left_MinCutBalDivSum_FAMean	0.00687	0.00320	0.17151
Left_MinCutBalDivSum_FiberLengthMean	0.23438	0.21147	0.01779
Left_MinCutBalDivSum_FiberN	0.13337	0.12011	0.00403 *
Left_MinCutBalDivSum_FiberNDivLength	0.11057	0.09321	0.00031 *
Left_MinCutBalDivSum_Unweighted	0.24513	0.22019	0.00206 *
Left_MinSpanningForest_FAMean	9.57924	9.06313	0.04242
Left_MinSpanningForest_FiberLengthMean	561.47024	560.36391	0.87722
Left_MinSpanningForest_FiberN	51.23077	53.73333	0.26795
Left_MinSpanningForest_FiberNDivLength	1.82447	1.89521	0.62729
Left_MinVertexCoverBinary_Unweighted	30.23077	29.73333	0.09601
Left_MinVertexCover_FAMean	9.23616	8.88642	0.01371
Left_MinVertexCover_FiberLengthMean	1064.27185	1027.73430	0.35926
Left_MinVertexCover_FiberN	1158.21154	1143.46667	0.55321
Left_MinVertexCover_FiberNDivLength	54.26322	51.17634	0.02122
Left_MinVertexCover_Unweighted	20.80769	20.83333	0.75017
Left_PGEigengap_FAMean	0.33446	0.29469	0.00215 *
Left_PGEigengap_FiberLengthMean	0.33383	0.29287	0.01329
Left_PGEigengap_FiberN	0.16980	0.15238	0.01654
Left_PGEigengap_FiberNDivLength	0.14486	0.13413	0.02837
Left_PGEigengap_Unweighted	0.30646	0.27160	0.00458 *
Left_Sum_FAMean	106.64056	96.80731	0.00056 *
Left_Sum_FiberLengthMean	8629.73791	8122.82646	0.13250
Left_Sum_FiberN	5514.61538	5049.73333	0.00000 *
Left_Sum_FiberNDivLength	233.06402	213.49323	0.00043 *
Left_Sum_Unweighted	282.50000	269.06667	0.00378 *
Right_AdjLMaxDivD_FAMean	1.32878	1.34242	0.14511
Right_AdjLMaxDivD_FiberLengthMean	1.39672	1.38478	0.30191
Right_AdjLMaxDivD_FiberN	2.00803	2.09048	0.05380
Right_AdjLMaxDivD_FiberNDivLength	1.76990	1.81343	0.09784
Right_AdjLMaxDivD_Unweighted	1.25268	1.24720	0.29540
Right_HoffmanBound_FAMean	4.47438	4.28666	0.00587 *
Right_HoffmanBound_FiberLengthMean	3.33823	3.39478	0.29902
Right_HoffmanBound_FiberN	2.67311	2.57701	0.05411
Right_HoffmanBound_FiberNDivLength	2.62635	2.48983	0.00560 *
Right_HoffmanBound_Unweighted	4.61480	4.50726	0.03806
Right_LogSpanningForestN_FAMean	52.25642	48.14346	0.00067 *
Right_LogSpanningForestN_FiberLengthMean	218.25106	216.24411	0.16431
Right_LogSpanningForestN_FiberN	190.62427	187.02757	0.00083 *
Right_LogSpanningForestN_FiberNDivLength	69.84080	66.17446	0.00022 *
Right_LogSpanningForestN_Unweighted	90.24090	88.51678	0.00541 *
Right_MinCutBalDivSum_FAMean	0.02476	0.00851	0.00496 *
Right_MinCutBalDivSum_FiberLengthMean	0.24577	0.22309	0.02216
Right_MinCutBalDivSum_FiberN	0.13346	0.12050	0.00346 *
Right_MinCutBalDivSum_FiberNDivLength	0.10831	0.09357	0.00072 *
Right_MinCutBalDivSum_Unweighted	0.23713	0.22022	0.01629
Right_MinSpanningForest_FAMean	10.30911	9.79708	0.10419
Right_MinSpanningForest_FiberLengthMean	532.13580	547.85331	0.00491 *
Right_MinSpanningForest_FiberN	50.76923	52.53333	0.26282
Right_MinSpanningForest_FiberNDivLength	1.94340	1.89232	0.58863
Right_MinVertexCoverBinary_Unweighted	29.07692	28.73333	0.15457
Right_MinVertexCover_FAMean	9.26572	9.03965	0.12382
Right_MinVertexCover_FiberLengthMean	934.26071	897.95882	0.23661
Right_MinVertexCover_FiberN	1169.63462	1122.93333	0.07986
Right_MinVertexCover_FiberNDivLength	53.57144	51.50298	0.10452
Right_MinVertexCover_Unweighted	20.11538	20.26667	0.10527
Right_PGEigengap_FAMean	0.32454	0.28808	0.00112 *
Right_PGEigengap_FiberLengthMean	0.34029	0.29461	0.00949 *
Right_PGEigengap_FiberN	0.17666	0.15912	0.02617
Right_PGEigengap_FiberNDivLength	0.15245	0.14034	0.01613
Right_PGEigengap_Unweighted	0.29582	0.26081	0.00087 *
Right_Sum_FAMean	105.62164	95.26436	0.00028 *
Right_Sum_FiberLengthMean	7644.90330	7086.91000	0.02974
Right_Sum_FiberN	5378.03846	4884.66667	0.00000 *
Right_Sum_FiberNDivLength	225.94776	206.97587	0.00018 *
Right_Sum_Unweighted	261.30769	248.26667	0.00019 *

**Table 3:** The graph-theoretic parameters computed for the 129-vertex graphs. The table contains their arithmetic means in the male and female groups, and the corresponding p-values in round 1 (see the “Statistical analysis” subsection). The results of the graph-parameters are defined in the caption of Table 1. Significant differences (*p* < 0.01) are denoted with an asterisk in the last column.

Property	Female	Male	p-value
All_AdjLMaxDivD_FAMean	1.40519	1.42604	0.10040
All_AdjLMaxDivD_FiberLengthMean	1.50483	1.50158	0.87806
All_AdjLMaxDivD_FiberN	2.14552	2.22254	0.15242
All_AdjLMaxDivD_FiberNDivLength	2.09783	2.04782	0.32031
All_AdjLMaxDivD_Unweighted	1.30028	1.29097	0.27278
All_HoffmanBound_FAMean	4.40157	4.29660	0.02644
All_HoffmanBound_FiberLengthMean	3.19684	3.24689	0.32568
All_HoffmanBound_FiberN	2.50604	2.48884	0.64956
All_HoffmanBound_FiberNDivLength	2.34647	2.41938	0.07720
All_HoffmanBound_Unweighted	4.62935	4.51267	0.01233
All_LogSpanningForestN_FAMean	194.37749	181.03525	0.00019 *
All_LogSpanningForestN_FiberLengthMean	739.78985	732.55388	0.09867
All_LogSpanningForestN_FiberN	599.76631	588.61699	0.00000 *
All_LogSpanningForestN_FiberNDivLength	210.52236	200.75240	0.00000 *
All_LogSpanningForestN_Unweighted	322.09324	316.62672	0.00218 *
All_MinCutBalDivSum_FAMean	0.00668	0.00324	0.05930
All_MinCutBalDivSum_FiberLengthMean	0.01706	0.01607	0.56293
All_MinCutBalDivSum_FiberN	0.02658	0.02429	0.26627
All_MinCutBalDivSum_FiberNDivLength	0.02495	0.02258	0.30029
All_MinCutBalDivSum_Unweighted	0.02218	0.02065	0.30082
All_MinSpanningForest_FAMean	30.14746	28.58509	0.02073
All_MinSpanningForest_FiberLengthMean	1642.68263	1664.23693	0.07510
All_MinSpanningForest_FiberN	140.23077	140.93333	0.55077
All_MinSpanningForest_FiberNDivLength	4.42401	4.43795	0.92181
All_MinVertexCoverBinary_Unweighted	96.46154	96.26667	0.66793
All_MinVertexCover_FAMean	29.56250	28.72424	0.02181
All_MinVertexCover_FiberLengthMean	3230.07900	3121.21684	0.29100
All_MinVertexCover_FiberN	2444.92308	2337.40000	0.00232 *
All_MinVertexCover_FiberNDivLength	120.18766	116.22553	0.02502
All_MinVertexCover_Unweighted	63.88462	63.96667	0.35805
All_PGEigengap_FAMean	0.03143	0.02928	0.25524
All_PGEigengap_FiberLengthMean	0.02427	0.02260	0.43054
All_PGEigengap_FiberN	0.02781	0.02453	0.01902
All_PGEigengap_FiberNDivLength	0.02880	0.02498	0.00792 *
All_PGEigengap_Unweighted	0.03012	0.02725	0.09661
All_Sum_FAMean	397.68878	360.50850	0.00015 *
All_Sum_FiberLengthMean	30670.09535	28478.19852	0.03582
All_Sum_FiberN	12375.61538	11458.13333	0.00000 *
All_Sum_FiberNDivLength	548.61301	510.71378	0.00008 *
All_Sum_Unweighted	1020.80769	972.86667	0.00026 *
Left_AdjLMaxDivD_FAMean	1.37823	1.39812	0.12792
Left_AdjLMaxDivD_FiberLengthMean	1.43638	1.42179	0.36739
Left_AdjLMaxDivD_FiberN	1.84672	1.92762	0.12247
Left_AdjLMaxDivD_FiberNDivLength	1.77313	1.80979	0.33521
Left_AdjLMaxDivD_Unweighted	1.26380	1.25501	0.16858
Left_HoffmanBound_FAMean	4.57539	4.44885	0.01512
Left_HoffmanBound_FiberLengthMean	3.23550	3.25088	0.77158
Left_HoffmanBound_FiberN	2.80373	2.74220	0.14090
Left_HoffmanBound_FiberNDivLength	2.70077	2.64308	0.21782
Left_HoffmanBound_Unweighted	4.75280	4.61941	0.00848 *
Left_LogSpanningForestN_FAMean	96.11000	89.25516	0.00106 *
Left_LogSpanningForestN_FiberLengthMean	373.09476	368.65582	0.08843
Left_LogSpanningForestN_FiberN	300.77613	295.83044	0.00014 *
Left_LogSpanningForestN_FiberNDivLength	105.01323	100.80980	0.00288 *
Left_LogSpanningForestN_Unweighted	162.01302	158.88026	0.01336
Left_MinCutBalDivSum_FAMean	0.00873	0.00273	0.05683
Left_MinCutBalDivSum_FiberLengthMean	0.19822	0.17378	0.00892 *
Left_MinCutBalDivSum_FiberN	0.12848	0.10467	0.00001 *
Left_MinCutBalDivSum_FiberNDivLength	0.06926	0.05546	0.00019 *
Left_MinCutBalDivSum_Unweighted	0.19535	0.17339	0.00265 *
Left_MinSpanningForest_FAMean	14.57467	13.88500	0.06189
Left_MinSpanningForest_FiberLengthMean	828.34729	834.54850	0.36946
Left_MinSpanningForest_FiberN	69.30769	72.20000	0.02902
Left_MinSpanningForest_FiberNDivLength	2.16989	2.25626	0.53695
Left_MinVertexCoverBinary_Unweighted	48.76923	48.86667	0.69355
Left_MinVertexCover_FAMean	14.65360	14.09857	0.01273
Left_MinVertexCover_FiberLengthMean	1700.29684	1637.18742	0.30481
Left_MinVertexCover_FiberN	1169.82692	1125.20000	0.06266
Left_MinVertexCover_FiberNDivLength	58.76113	56.23736	0.06303
Left_MinVertexCover_Unweighted	32.28846	32.30000	0.88865
Left_PGEigengap_FAMean	0.22611	0.19656	0.00995 *
Left_PGEigengap_FiberLengthMean	0.23241	0.20065	0.02197
Left_PGEigengap_FiberN	0.12346	0.10569	0.00382 *
Left_PGEigengap_FiberNDivLength	0.09689	0.08572	0.00223 *
Left_PGEigengap_Unweighted	0.20204	0.17516	0.01081
Left_Sum_FAMean	197.41850	178.80563	0.00032 *
Left_Sum_FiberLengthMean	16079.40944	14931.40760	0.07487
Left_Sum_FiberN	6071.96154	5641.93333	0.00000 *
Left_Sum_FiberNDivLength	269.09760	251.40080	0.00100 *
Left_Sum_Unweighted	519.53846	492.86667	0.00232 *
Right_AdjLMaxDivD_FAMean	1.35746	1.36837	0.36353
Right_AdjLMaxDivD_FiberLengthMean	1.42015	1.41129	0.54264
Right_AdjLMaxDivD_FiberN	2.05564	2.19134	0.01338
Right_AdjLMaxDivD_FiberNDivLength	1.82146	1.86716	0.20816
Right_AdjLMaxDivD_Unweighted	1.26684	1.25522	0.12057
Right_HoffmanBound_FAMean	4.37886	4.29574	0.20294
Right_HoffmanBound_FiberLengthMean	3.32686	3.36662	0.49418
Right_HoffmanBound_FiberN	2.66511	2.56838	0.01727
Right_HoffmanBound_FiberNDivLength	2.68679	2.59830	0.01992
Right_HoffmanBound_Unweighted	4.60861	4.51407	0.08448
Right_LogSpanningForestN_FAMean	93.41904	87.28295	0.00143 *
Right_LogSpanningForestN_FiberLengthMean	358.00491	354.73456	0.14280
Right_LogSpanningForestN_FiberN	291.08563	285.72242	0.00045 *
Right_LogSpanningForestN_FiberNDivLength	100.74383	96.22891	0.00051 *
Right_LogSpanningForestN_Unweighted	154.36558	151.96595	0.01158
Right_MinCutBalDivSum_FAMean	0.02361	0.01005	0.00807 *
Right_MinCutBalDivSum_FiberLengthMean	0.20000	0.17303	0.00768 *
Right_MinCutBalDivSum_FiberN	0.11452	0.10111	0.00563 *
Right_MinCutBalDivSum_FiberNDivLength	0.06865	0.06326	0.09375
Right_MinCutBalDivSum_Unweighted	0.19180	0.16911	0.00492 *
Right_MinSpanningForest_FAMean	15.61479	14.88977	0.06537
Right_MinSpanningForest_FiberLengthMean	808.14079	824.37649	0.03729
Right_MinSpanningForest_FiberN	70.46154	68.93333	0.07096
Right_MinSpanningForest_FiberNDivLength	2.32813	2.26810	0.46298
Right_MinVertexCoverBinary_Unweighted	47.34615	47.00000	0.29760
Right_MinVertexCover_FAMean	14.70648	14.40974	0.13709
Right_MinVertexCover_FiberLengthMean	1516.99670	1461.52391	0.23679
Right_MinVertexCover_FiberN	1175.50000	1166.36667	0.68666
Right_MinVertexCover_FiberNDivLength	59.59421	58.78162	0.47843
Right_MinVertexCover_Unweighted	31.61538	31.73333	0.20363
Right_PGEigengap_FAMean	0.22838	0.19627	0.00296 *
Right_PGEigengap_FiberLengthMean	0.23840	0.19868	0.01013
Right_PGEigengap_FiberN	0.12500	0.11049	0.00869 *
Right_PGEigengap_FiberNDivLength	0.10075	0.09371	0.03033
Right_PGEigengap_Unweighted	0.20584	0.17429	0.00242 *
Right_Sum_FAMean	190.48228	172.48988	0.00062 *
Right_Sum_FiberLengthMean	13952.01182	13003.32443	0.04620
Right_Sum_FiberN	5935.73077	5525.26667	0.00001 *
Right_Sum_FiberNDivLength	262.31420	246.32048	0.00180 *
Right_Sum_Unweighted	477.38462	454.86667	0.00068 *

**Table 4:** The graph-theoretic parameters computed for the 234-vertex graphs. The table contains their arithmetic means in the male and female groups, and the corresponding p-values in round 1 (see the “Statistical analysis” subsection). The results of the graph-parameters are defined in the caption of Table 1. Significant differences (*p* < 0.01) are denoted with an asterisk in the last column.

Property	Female	Male	p-value
All_AdjLMaxDivD_FAMean	1.59824	1.62177	0.20251
All_AdjLMaxDivD_FiberLengthMean	1.72504	1.73145	0.81358
All_AdjLMaxDivD_FiberN	3.00790	2.99029	0.82198
All_AdjLMaxDivD_FiberNDivLength	3.06003	2.88353	0.06518
All_AdjLMaxDivD_Unweighted	1.44314	1.43766	0.65566
All_HoffmanBound_FAMean	4.11594	4.07125	0.25943
All_HoffmanBound_FiberLengthMean	3.09745	3.17400	0.10768
All_HoffmanBound_FiberN	2.33189	2.36477	0.36494
All_HoffmanBound_FiberNDivLength	2.24918	2.30019	0.09467
All_HoffmanBound_Unweighted	4.26449	4.22883	0.31200
All_LogSpanningForestN_FAMean	333.13181	309.36644	0.00043 *
All_LogSpanningForestN_FiberLengthMean	1319.41755	1305.88683	0.09486
All_LogSpanningForestN_FiberN	958.26596	942.51022	0.00091 *
All_LogSpanningForestN_FiberNDivLength	261.96128	250.78897	0.00106 *
All_LogSpanningForestN_Unweighted	575.69684	565.48272	0.01073
All_MinCutBalDivSum_FAMean	0.00258	0.00069	0.03188
All_MinCutBalDivSum_FiberLengthMean	0.01055	0.00997	0.56364
All_MinCutBalDivSum_FiberN	0.02503	0.02259	0.20939
All_MinCutBalDivSum_FiberNDivLength	0.02020	0.01710	0.03711
All_MinCutBalDivSum_Unweighted	0.01316	0.01222	0.25998
All_MinSpanningForest_FAMean	51.00196	48.57885	0.01194
All_MinSpanningForest_FiberLengthMean	2804.74772	2831.83990	0.06357
All_MinSpanningForest_FiberN	245.92308	245.00000	0.53375
All_MinSpanningForest_FiberNDivLength	7.99545	7.91594	0.74227
All_MinVertexCoverBinary_Unweighted	166.65385	165.40000	0.14781
All_MinVertexCover_FAMean	51.98307	50.16832	0.00289 *
All_MinVertexCover_FiberLengthMean	5248.81515	5090.58422	0.34139
All_MinVertexCover_FiberN	2430.57692	2326.23333	0.00055 *
All_MinVertexCover_FiberNDivLength	127.46338	125.41222	0.14913
All_MinVertexCover_Unweighted	116.23077	116.26667	0.79591
All_PGEigengap_FAMean	0.01892	0.01744	0.20099
All_PGEigengap_FiberLengthMean	0.01499	0.01383	0.36831
All_PGEigengap_FiberN	0.02517	0.02182	0.00826 *
All_PGEigengap_FiberNDivLength	0.02453	0.02090	0.00321 *
All_PGEigengap_Unweighted	0.01750	0.01579	0.09480
All_Sum_FAMean	689.73851	628.62387	0.00014 *
All_Sum_FiberLengthMean	51558.63408	48397.55225	0.05764
All_Sum_FiberN	13267.88462	12438.86667	0.00000 *
All_Sum_FiberNDivLength	618.33865	586.27221	0.00044 *
All_Sum_Unweighted	1826.03846	1742.66667	0.00063 *
Left_AdjLMaxDivD_FAMean	1.58597	1.61184	0.19191
Left_AdjLMaxDivD_FiberLengthMean	1.67378	1.67488	0.96164
Left_AdjLMaxDivD_FiberN	2.51455	2.59709	0.24706
Left_AdjLMaxDivD_FiberNDivLength	2.46008	2.44949	0.86194
Left_AdjLMaxDivD_Unweighted	1.42058	1.41216	0.45842
Left_HoffmanBound_FAMean	4.19268	4.14961	0.31372
Left_HoffmanBound_FiberLengthMean	3.12191	3.15801	0.48134
Left_HoffmanBound_FiberN	2.63594	2.59362	0.26584
Left_HoffmanBound_FiberNDivLength	2.52966	2.50832	0.61082
Left_HoffmanBound_Unweighted	4.35117	4.29447	0.14153
Left_LogSpanningForestN_FAMean	164.44676	151.96676	0.00060 *
Left_LogSpanningForestN_FiberLengthMean	670.03055	661.91968	0.08435
Left_LogSpanningForestN_FiberN	484.10215	477.60923	0.02239
Left_LogSpanningForestN_FiberNDivLength	130.53658	126.33570	0.07747
Left_LogSpanningForestN_Unweighted	290.55966	285.58194	0.03117
Left_MinCutBalDivSum_FAMean	0.00188	0.00000	0.01777
Left_MinCutBalDivSum_FiberLengthMean	0.14735	0.12215	0.00411 *
Left_MinCutBalDivSum_FiberN	0.10539	0.08507	0.00002 *
Left_MinCutBalDivSum_FiberNDivLength	0.02918	0.02209	0.00642 *
Left_MinCutBalDivSum_Unweighted	0.14392	0.12444	0.00154 *
Left_MinSpanningForest_FAMean	25.10810	23.82569	0.01171
Left_MinSpanningForest_FiberLengthMean	1431.81175	1435.42334	0.67083
Left_MinSpanningForest_FiberN	126.92308	126.06667	0.61065
Left_MinSpanningForest_FiberNDivLength	4.18418	4.03231	0.41157
Left_MinVertexCoverBinary_Unweighted	84.00000	83.20000	0.15412
Left_MinVertexCover_FAMean	25.89765	24.77675	0.00107 *
Left_MinVertexCover_FiberLengthMean	2746.38841	2650.87601	0.30587
Left_MinVertexCover_FiberN	1197.30769	1175.86667	0.33225
Left_MinVertexCover_FiberNDivLength	65.03508	64.53534	0.69540
Left_MinVertexCover_Unweighted	59.15385	59.13333	0.87392
Left_PGEigengap_FAMean	0.14563	0.12266	0.00141 *
Left_PGEigengap_FiberLengthMean	0.14891	0.12366	0.00554 *
Left_PGEigengap_FiberN	0.09767	0.08037	0.00066 *
Left_PGEigengap_FiberNDivLength	0.07383	0.06273	0.00013 *
Left_PGEigengap_Unweighted	0.12909	0.10748	0.00176 *
Left_Sum_FAMean	341.86488	310.44231	0.00026 *
Left_Sum_FiberLengthMean	26855.84149	24971.13564	0.06460
Left_Sum_FiberN	6551.88462	6204.20000	0.00040 *
Left_Sum_FiberNDivLength	306.39045	293.36221	0.01442
Left_Sum_Unweighted	926.34615	881.53333	0.00293 *
Right_AdjLMaxDivD_FAMean	1.51832	1.54700	0.12507
Right_AdjLMaxDivD_FiberLengthMean	1.61182	1.62906	0.37469
Right_AdjLMaxDivD_FiberN	2.57025	2.75485	0.02819
Right_AdjLMaxDivD_FiberNDivLength	2.34753	2.37750	0.61484
Right_AdjLMaxDivD_Unweighted	1.39063	1.39160	0.92921
Right_HoffmanBound_FAMean	4.12472	4.11339	0.81603
Right_HoffmanBound_FiberLengthMean	3.21816	3.32010	0.04777
Right_HoffmanBound_FiberN	2.54183	2.48873	0.06857
Right_HoffmanBound_FiberNDivLength	2.55201	2.50267	0.17018
Right_HoffmanBound_Unweighted	4.31441	4.30433	0.78744
Right_LogSpanningForestN_FAMean	163.64781	152.84474	0.00380 *
Right_LogSpanningForestN_FiberLengthMean	640.52307	635.23398	0.18037
Right_LogSpanningForestN_FiberN	465.97727	459.03694	0.01282
Right_LogSpanningForestN_FiberNDivLength	126.61017	120.67006	0.00940 *
Right_LogSpanningForestN_Unweighted	279.20359	274.72648	0.03170
Right_MinCutBalDivSum_FAMean	0.00959	0.00322	0.03000
Right_MinCutBalDivSum_FiberLengthMean	0.14992	0.12148	0.00234 *
Right_MinCutBalDivSum_FiberN	0.10001	0.08633	0.00234 *
Right_MinCutBalDivSum_FiberNDivLength	0.03193	0.02816	0.14205
Right_MinCutBalDivSum_Unweighted	0.13748	0.11683	0.00462 *
Right_MinSpanningForest_FAMean	25.95958	24.86484	0.05085
Right_MinSpanningForest_FiberLengthMean	1367.27500	1390.89912	0.00601 *
Right_MinSpanningForest_FiberN	119.19231	118.86667	0.70146
Right_MinSpanningForest_FiberNDivLength	3.93775	3.96494	0.81094
Right_MinVertexCoverBinary_Unweighted	82.30769	81.66667	0.20770
Right_MinVertexCover_FAMean	25.90289	25.23516	0.03478
Right_MinVertexCover_FiberLengthMean	2485.78834	2418.73395	0.39406
Right_MinVertexCover_FiberN	1128.59615	1112.30000	0.37231
Right_MinVertexCover_FiberNDivLength	60.30418	59.94168	0.76940
Right_MinVertexCover_Unweighted	57.07692	57.16667	0.40299
Right_PGEigengap_FAMean	0.14077	0.11639	0.00074 *
Right_PGEigengap_FiberLengthMean	0.15024	0.12064	0.00367 *
Right_PGEigengap_FiberN	0.09544	0.07733	0.00009 *
Right_PGEigengap_FiberNDivLength	0.07248	0.06304	0.00041 *
Right_PGEigengap_Unweighted	0.12293	0.10002	0.00095 *
Right_Sum_FAMean	337.74022	308.44201	0.00085 *
Right_Sum_FiberLengthMean	24086.28238	22847.27016	0.12171
Right_Sum_FiberN	6343.42308	5974.26667	0.00012 *
Right_Sum_FiberNDivLength	294.65559	280.13740	0.00562 *
Right_Sum_Unweighted	874.00000	835.60000	0.00224 *

**Table 5:** The graph-theoretic parameters computed for the 463-vertex graphs. The table contains their arithmetic means in the male and female groups, and the corresponding p-values in round 1 (see the “Statistical analysis” subsection). The results of the graph-parameters are defined in the caption of Table 1. Significant differences (*p* < 0.01) are denoted with an asterisk in the last column.

Property	Female	Male	p-value
All_AdjLMaxDivD_FAMean	2.15050	2.14489	0.86385
All_AdjLMaxDivD_FiberLengthMean	2.35868	2.34695	0.80876
All_AdjLMaxDivD_FiberN	5.14838	5.00652	0.35870
All_AdjLMaxDivD_FiberNDivLength	5.17072	4.78287	0.02543
All_AdjLMaxDivD_Unweighted	1.89062	1.84578	0.06482
All_HoffmanBound_FAMean	3.63940	3.62013	0.57408
All_HoffmanBound_FiberLengthMean	2.92490	2.98466	0.17340
All_HoffmanBound_FiberN	2.23619	2.26557	0.30055
All_HoffmanBound_FiberNDivLength	2.20178	2.23871	0.13550
All_HoffmanBound_Unweighted	3.73661	3.72935	0.82472
All_LogSpanningForestN_FAMean	446.86116	416.54482	0.03232
All_LogSpanningForestN_FiberLengthMean	2324.68381	2325.52712	0.96824
All_LogSpanningForestN_FiberN	1456.24015	1445.53700	0.36683
All_LogSpanningForestN_FiberNDivLength	149.01647	138.16817	0.15229
All_LogSpanningForestN_Unweighted	942.01654	944.27877	0.83734
All_MinCutBalDivSum_FAMean	0.00000	0.00000	nan
All_MinCutBalDivSum_FiberLengthMean	0.00769	0.00723	0.57442
All_MinCutBalDivSum_FiberN	0.02405	0.02168	0.21132
All_MinCutBalDivSum_FiberNDivLength	0.00000	0.00000	0.45008
All_MinCutBalDivSum_Unweighted	0.00898	0.00834	0.32475
All_MinSpanningForest_FAMean	98.19730	92.47667	0.00151 *
All_MinSpanningForest_FiberLengthMean	5358.83904	5379.38212	0.44199
All_MinSpanningForest_FiberN	481.46154	479.20000	0.45787
All_MinSpanningForest_FiberNDivLength	18.53246	18.36575	0.71037
All_MinVertexCoverBinary_Unweighted	276.15385	280.33333	0.12225
All_MinVertexCover_FAMean	89.53747	87.25805	0.06974
All_MinVertexCover_FiberLengthMean	8136.04292	7957.20990	0.48358
All_MinVertexCover_FiberN	2430.61538	2344.50000	0.00056 *
All_MinVertexCover_FiberNDivLength	129.82332	126.64639	0.02087
All_MinVertexCover_Unweighted	222.57692	223.33333	0.39844
All_PGEigengap_FAMean	0.01106	0.01201	0.54543
All_PGEigengap_FiberLengthMean	0.00860	0.00960	0.45409
All_PGEigengap_FiberN	0.01894	0.01927	0.89543
All_PGEigengap_FiberNDivLength	0.01773	0.01767	0.97772
All_PGEigengap_Unweighted	0.00995	0.01067	0.59117
All_Sum_FAMean	1033.36931	961.08503	0.00297 *
All_Sum_FiberLengthMean	74747.99556	71461.78993	0.18467
All_Sum_FiberN	13609.34615	12823.40000	0.00000 *
All_Sum_FiberNDivLength	652.17760	623.38731	0.00139 *
All_Sum_Unweighted	2801.69231	2746.20000	0.21290
Left_AdjLMaxDivD_FAMean	2.14627	2.14335	0.93401
Left_AdjLMaxDivD_FiberLengthMean	2.29338	2.29214	0.97718
Left_AdjLMaxDivD_FiberN	4.03186	4.16381	0.29128
Left_AdjLMaxDivD_FiberNDivLength	3.93717	3.84897	0.38654
Left_AdjLMaxDivD_Unweighted	1.86339	1.81508	0.04174
Left_HoffmanBound_FAMean	3.74670	3.77335	0.55549
Left_HoffmanBound_FiberLengthMean	2.94312	2.99233	0.25660
Left_HoffmanBound_FiberN	2.51168	2.47461	0.28318
Left_HoffmanBound_FiberNDivLength	2.44470	2.45140	0.85286
Left_HoffmanBound_Unweighted	3.82814	3.84621	0.65499
Left_LogSpanningForestN_FAMean	212.18613	197.08273	0.04326
Left_LogSpanningForestN_FiberLengthMean	1159.44274	1165.33847	0.58696
Left_LogSpanningForestN_FiberN	723.10349	723.01322	0.98899
Left_LogSpanningForestN_FiberNDivLength	70.44766	65.77187	0.31060
Left_LogSpanningForestN_Unweighted	467.24325	470.94213	0.52729
Left_MinCutBalDivSum_FAMean	0.00000	0.00000	nan
Left_MinCutBalDivSum_FiberLengthMean	0.09355	0.07667	0.00655 *
Left_MinCutBalDivSum_FiberN	0.07158	0.05914	0.00062 *
Left_MinCutBalDivSum_FiberNDivLength	0.00000	0.00000	nan
Left_MinCutBalDivSum_Unweighted	0.09416	0.07896	0.00153 *
Left_MinSpanningForest_FAMean	47.28302	44.78250	0.00239 *
Left_MinSpanningForest_FiberLengthMean	2702.23206	2712.65026	0.49327
Left_MinSpanningForest_FiberN	244.11538	244.46667	0.89014
Left_MinSpanningForest_FiberNDivLength	9.45842	9.50259	0.88229
Left_MinVertexCoverBinary_Unweighted	137.19231	140.00000	0.06105
Left_MinVertexCover_FAMean	43.50481	42.59720	0.16942
Left_MinVertexCover_FiberLengthMean	4136.87086	4052.71473	0.55895
Left_MinVertexCover_FiberN	1168.19231	1153.66667	0.46021
Left_MinVertexCover_FiberNDivLength	63.94002	64.04107	0.92511
Left_MinVertexCover_Unweighted	111.38462	112.26667	0.09259
Left_PGEigengap_FAMean	0.08402	0.07554	0.28777
Left_PGEigengap_FiberLengthMean	0.08669	0.07722	0.29463
Left_PGEigengap_FiberN	0.06812	0.05737	0.09675
Left_PGEigengap_FiberNDivLength	0.05084	0.04481	0.18106
Left_PGEigengap_Unweighted	0.07190	0.06398	0.24844
Left_Sum_FAMean	504.02280	470.30921	0.01077
Left_Sum_FiberLengthMean	38178.70022	36255.83071	0.19037
Left_Sum_FiberN	6716.53846	6389.20000	0.00107 *
Left_Sum_FiberNDivLength	322.55630	311.23280	0.04079
Left_Sum_Unweighted	1401.80769	1380.33333	0.39428
Right_AdjLMaxDivD_FAMean	2.00996	2.02718	0.61502
Right_AdjLMaxDivD_FiberLengthMean	2.15381	2.18170	0.41400
Right_AdjLMaxDivD_FiberN	4.11898	4.41926	0.03397
Right_AdjLMaxDivD_FiberNDivLength	3.79534	3.75488	0.70781
Right_AdjLMaxDivD_Unweighted	1.79189	1.77141	0.38704
Right_HoffmanBound_FAMean	3.63008	3.59884	0.45778
Right_HoffmanBound_FiberLengthMean	3.00591	3.02300	0.69490
Right_HoffmanBound_FiberN	2.40837	2.33314	0.00150 *
Right_HoffmanBound_FiberNDivLength	2.45857	2.38848	0.01602
Right_HoffmanBound_Unweighted	3.71704	3.69299	0.50645
Right_LogSpanningForestN_FAMean	228.90719	215.28259	0.07936
Right_LogSpanningForestN_FiberLengthMean	1154.04516	1148.91122	0.63377
Right_LogSpanningForestN_FiberN	724.05083	716.03208	0.22608
Right_LogSpanningForestN_FiberNDivLength	72.92465	68.45678	0.30478
Right_LogSpanningForestN_Unweighted	467.61765	466.56728	0.85195
Right_MinCutBalDivSum_FAMean	0.00050	0.00000	0.19303
Right_MinCutBalDivSum_FiberLengthMean	0.10021	0.08439	0.01271
Right_MinCutBalDivSum_FiberN	0.07599	0.06701	0.00641 *
Right_MinCutBalDivSum_FiberNDivLength	0.00034	0.00000	0.18042
Right_MinCutBalDivSum_Unweighted	0.09573	0.08171	0.01034
Right_MinSpanningForest_FAMean	50.98056	47.79220	0.00435 *
Right_MinSpanningForest_FiberLengthMean	2655.83115	2655.71544	0.99483
Right_MinSpanningForest_FiberN	238.96154	236.00000	0.15420
Right_MinSpanningForest_FiberNDivLength	9.28191	9.00082	0.18645
Right_MinVertexCoverBinary_Unweighted	138.30769	140.00000	0.25603
Right_MinVertexCover_FAMean	45.80119	44.57707	0.07765
Right_MinVertexCover_FiberLengthMean	3994.00115	3884.90036	0.36802
Right_MinVertexCover_FiberN	1144.80769	1129.73333	0.41752
Right_MinVertexCover_FiberNDivLength	62.35579	61.50301	0.47854
Right_MinVertexCover_Unweighted	111.09615	111.10000	0.99385
Right_PGEigengap_FAMean	0.08312	0.07683	0.33378
Right_PGEigengap_FiberLengthMean	0.08538	0.07887	0.40909
Right_PGEigengap_FiberN	0.06631	0.06080	0.28067
Right_PGEigengap_FiberNDivLength	0.05084	0.04854	0.52890
Right_PGEigengap_Unweighted	0.07102	0.06430	0.25554
Right_Sum_FAMean	517.36095	481.68012	0.00745 *
Right_Sum_FiberLengthMean	35857.03890	34486.76733	0.26347
Right_Sum_FiberN	6524.53846	6187.46667	0.00050 *
Right_Sum_FiberNDivLength	312.50248	299.09835	0.01170
Right_Sum_Unweighted	1368.00000	1339.06667	0.20464

**Table 6:** The graph-theoretic parameters computed for the 1015-vertex graphs. The table contains their arithmetic means in the male and female groups, and the corresponding p-values in round 1 (see the “Statistical analysis” subsection). The results of the graph-parameters are defined in the caption of Table 1. Significant differences (*p* < 0.01) are denoted with an asterisk in the last column.

Property	Female	Male	p-value
All_AdjLMaxDivD_FAMean	3.26830	3.22666	0.50479
All_AdjLMaxDivD_FiberLengthMean	3.62257	3.59299	0.70455
All_AdjLMaxDivD_FiberN	10.28187	9.77558	0.14303
All_AdjLMaxDivD_FiberNDivLength	10.27862	9.34497	0.01310
All_AdjLMaxDivD_Unweighted	2.82618	2.73441	0.05794
All_HoffmanBound_FAMean	3.14150	3.12055	0.49395
All_HoffmanBound_FiberLengthMean	2.70017	2.72337	0.56140
All_HoffmanBound_FiberN	2.17344	2.19541	0.33617
All_HoffmanBound_FiberNDivLength	2.16670	2.18823	0.28867
All_HoffmanBound_Unweighted	3.14485	3.17447	0.28362
All_LogSpanningForestN_FAMean	462.87895	407.81850	0.01598
All_LogSpanningForestN_FiberLengthMean	4026.79612	4064.06908	0.51373
All_LogSpanningForestN_FiberN	2113.44981	2111.38514	0.93474
All_LogSpanningForestN_FiberNDivLength	-360.95367	-395.04681	0.00224 *
All_LogSpanningForestN_Unweighted	1442.82519	1456.54027	0.56229
All_MinCutBalDivSum_FAMean	0.00000	0.00000	nan
All_MinCutBalDivSum_FiberLengthMean	0.00592	0.00549	0.50112
All_MinCutBalDivSum_FiberN	0.02378	0.02125	0.17485
All_MinCutBalDivSum_FiberNDivLength	0.00000	0.00000	nan
All_MinCutBalDivSum_Unweighted	0.00670	0.00622	0.35913
All_MinSpanningForest_FAMean	202.01934	194.58409	0.03795
All_MinSpanningForest_FiberLengthMean	10853.72303	10980.29025	0.33121
All_MinSpanningForest_FiberN	949.84615	961.20000	0.14601
All_MinSpanningForest_FiberNDivLength	42.55517	43.29246	0.28142
All_MinVertexCoverBinary_Unweighted	455.07692	465.86667	0.10986
All_MinVertexCover_FAMean	152.17142	149.81916	0.35180
All_MinVertexCover_FiberLengthMean	12543.46037	12370.61833	0.67404
All_MinVertexCover_FiberN	2511.09615	2425.46667	0.00196 *
All_MinVertexCover_FiberNDivLength	137.10003	134.93973	0.07471
All_MinVertexCover_Unweighted	416.57692	424.80000	0.08280
All_PGEigengap_FAMean	0.00000	0.00000	0.05029
All_PGEigengap_FiberLengthMean	0.00000	0.00000	0.02339
All_PGEigengap_FiberN	0.00000	0.00000	0.45872
All_PGEigengap_FiberNDivLength	0.00000	0.00000	0.21400
All_PGEigengap_Unweighted	0.00000	0.00000	0.41265
All_Sum_FAMean	1459.55405	1376.26719	0.01542
All_Sum_FiberLengthMean	102370.95810	98486.06872	0.25420
All_Sum_FiberN	13782.73077	13029.33333	0.00000 *
All_Sum_FiberNDivLength	673.10973	647.37263	0.00311 *
All_Sum_Unweighted	3997.69231	3963.53333	0.62288
Left_AdjLMaxDivD_FAMean	3.21004	3.16445	0.48865
Left_AdjLMaxDivD_FiberLengthMean	3.47972	3.46192	0.80613
Left_AdjLMaxDivD_FiberN	7.44128	7.62750	0.44388
Left_AdjLMaxDivD_FiberNDivLength	7.46716	7.12690	0.10215
Left_AdjLMaxDivD_Unweighted	2.74415	2.65103	0.05546
Left_HoffmanBound_FAMean	3.18230	3.21579	0.35116
Left_HoffmanBound_FiberLengthMean	2.71658	2.72868	0.77232
Left_HoffmanBound_FiberN	2.39866	2.35113	0.07634
Left_HoffmanBound_FiberNDivLength	2.35921	2.37377	0.61676
Left_HoffmanBound_Unweighted	3.18178	3.22401	0.15588
Left_LogSpanningForestN_FAMean	218.88060	190.32522	0.02158
Left_LogSpanningForestN_FiberLengthMean	2013.63413	2042.75930	0.33131
Left_LogSpanningForestN_FiberN	1055.88675	1060.69344	0.74503
Left_LogSpanningForestN_FiberNDivLength	-176.77031	-199.57564	0.00208 *
Left_LogSpanningForestN_Unweighted	721.11018	732.40889	0.38115
Left_MinCutBalDivSum_FAMean	0.00000	0.00000	nan
Left_MinCutBalDivSum_FiberLengthMean	0.05605	0.04820	0.04349
Left_MinCutBalDivSum_FiberN	0.04732	0.03987	0.00320 *
Left_MinCutBalDivSum_FiberNDivLength	0.00000	0.00000	nan
Left_MinCutBalDivSum_Unweighted	0.05656	0.04724	0.00844 *
Left_MinSpanningForest_FAMean	97.75051	94.30097	0.04097
Left_MinSpanningForest_FiberLengthMean	5423.31192	5502.54221	0.28149
Left_MinSpanningForest_FiberN	478.19231	483.26667	0.38055
Left_MinSpanningForest_FiberNDivLength	21.27865	21.70041	0.29800
Left_MinVertexCoverBinary_Unweighted	227.11538	233.73333	0.07202
Left_MinVertexCover_FAMean	74.41098	73.74517	0.62229
Left_MinVertexCover_FiberLengthMean	6392.76727	6305.67989	0.71160
Left_MinVertexCover_FiberN	1201.75000	1192.60000	0.61270
Left_MinVertexCover_FiberNDivLength	67.50503	67.58761	0.93909
Left_MinVertexCover_Unweighted	207.92308	212.90000	0.03594
Left_PGEigengap_FAMean	0.01517	0.00869	0.39805
Left_PGEigengap_FiberLengthMean	0.01664	0.00955	0.40077
Left_PGEigengap_FiberN	0.01331	0.00759	0.39605
Left_PGEigengap_FiberNDivLength	0.00973	0.00575	0.42023
Left_PGEigengap_Unweighted	0.01318	0.00736	0.37961
Left_Sum_FAMean	717.28030	678.01829	0.04387
Left_Sum_FiberLengthMean	52657.14323	50331.86577	0.26591
Left_Sum_FiberN	6802.46154	6493.86667	0.00199 *
Left_Sum_FiberNDivLength	333.23344	323.69931	0.08560
Left_Sum_Unweighted	2011.69231	2008.26667	0.93270
Right_AdjLMaxDivD_FAMean	3.09017	3.11666	0.66565
Right_AdjLMaxDivD_FiberLengthMean	3.38484	3.44122	0.35126
Right_AdjLMaxDivD_FiberN	7.59635	8.17470	0.02932
Right_AdjLMaxDivD_FiberNDivLength	7.15573	7.00011	0.45723
Right_AdjLMaxDivD_Unweighted	2.71176	2.67597	0.42264
Right_HoffmanBound_FAMean	3.13585	3.08090	0.11839
Right_HoffmanBound_FiberLengthMean	2.73005	2.72344	0.86660
Right_HoffmanBound_FiberN	2.32876	2.24940	0.00046 *
Right_HoffmanBound_FiberNDivLength	2.37433	2.30135	0.00443 *
Right_HoffmanBound_Unweighted	3.14286	3.12279	0.54207
Right_LogSpanningForestN_FAMean	235.40566	211.61663	0.05014
Right_LogSpanningForestN_FiberLengthMean	1998.88318	2008.43828	0.72711
Right_LogSpanningForestN_FiberN	1046.99698	1041.17496	0.64908
Right_LogSpanningForestN_FiberNDivLength	-189.47349	-199.15924	0.14329
Right_LogSpanningForestN_Unweighted	712.41597	715.86196	0.76515
Right_MinCutBalDivSum_FAMean	0.00000	0.00000	nan
Right_MinCutBalDivSum_FiberLengthMean	0.06040	0.05337	0.07185
Right_MinCutBalDivSum_FiberN	0.05063	0.04341	0.00046 *
Right_MinCutBalDivSum_FiberNDivLength	0.00000	0.00000	nan
Right_MinCutBalDivSum_Unweighted	0.06023	0.05239	0.02254
Right_MinSpanningForest_FAMean	104.45182	100.51343	0.04268
Right_MinSpanningForest_FiberLengthMean	5417.44253	5459.18134	0.50797
Right_MinSpanningForest_FiberN	475.53846	481.20000	0.17743
Right_MinSpanningForest_FiberNDivLength	21.44891	21.86007	0.30998
Right_MinVertexCoverBinary_Unweighted	227.19231	231.46667	0.21896
Right_MinVertexCover_FAMean	77.44842	76.18852	0.30232
Right_MinVertexCover_FiberLengthMean	6145.06803	6019.33720	0.51427
Right_MinVertexCover_FiberN	1186.23077	1171.70000	0.38726
Right_MinVertexCover_FiberNDivLength	65.73066	65.63902	0.92812
Right_MinVertexCover_Unweighted	208.23077	211.36667	0.22097
Right_PGEigengap_FAMean	0.01030	0.00304	0.24753
Right_PGEigengap_FiberLengthMean	0.01095	0.00224	0.18252
Right_PGEigengap_FiberN	0.00757	0.00240	0.26739
Right_PGEigengap_FiberNDivLength	0.00604	0.00203	0.28138
Right_PGEigengap_Unweighted	0.00857	0.00245	0.24098
Right_Sum_FAMean	727.59000	688.66472	0.02233
Right_Sum_FiberLengthMean	48923.40412	47444.32994	0.34244
Right_Sum_FiberN	6610.34615	6288.26667	0.00073 *
Right_Sum_FiberNDivLength	322.60919	310.44886	0.01780
Right_Sum_Unweighted	1949.00000	1933.46667	0.63034

It is known that there are statistical differences in the size and the weight of the female and the male cerebra [18]. It was also published [19] that female brains statistically have a smaller gray matter/white matter ratio, that is, a higher white matter/gray matter ratio than male brains. We argue that this observation is in line with the quantitative differences in the fibers and edges in the connectomes of the sexes: In a simplified view, the edges of the braingraph correspond to the fibers of the myelinated axons in the white matter, while the nodes of the graph to areas of the gray matter. Therefore, since females have a higher white matter/gray matter ratio than males by [19] that fact implies that the number of detected fibers by the tractography step of the processing is relatively higher in females than in males, and this higher number of fibers imply higher number of edges in female connectomes.

We are carefully dealing with the possibilities of artifacts in the edge number differences in the “Methods” section.

### Minimum cut and balanced minimum cut

**Fig 1:**
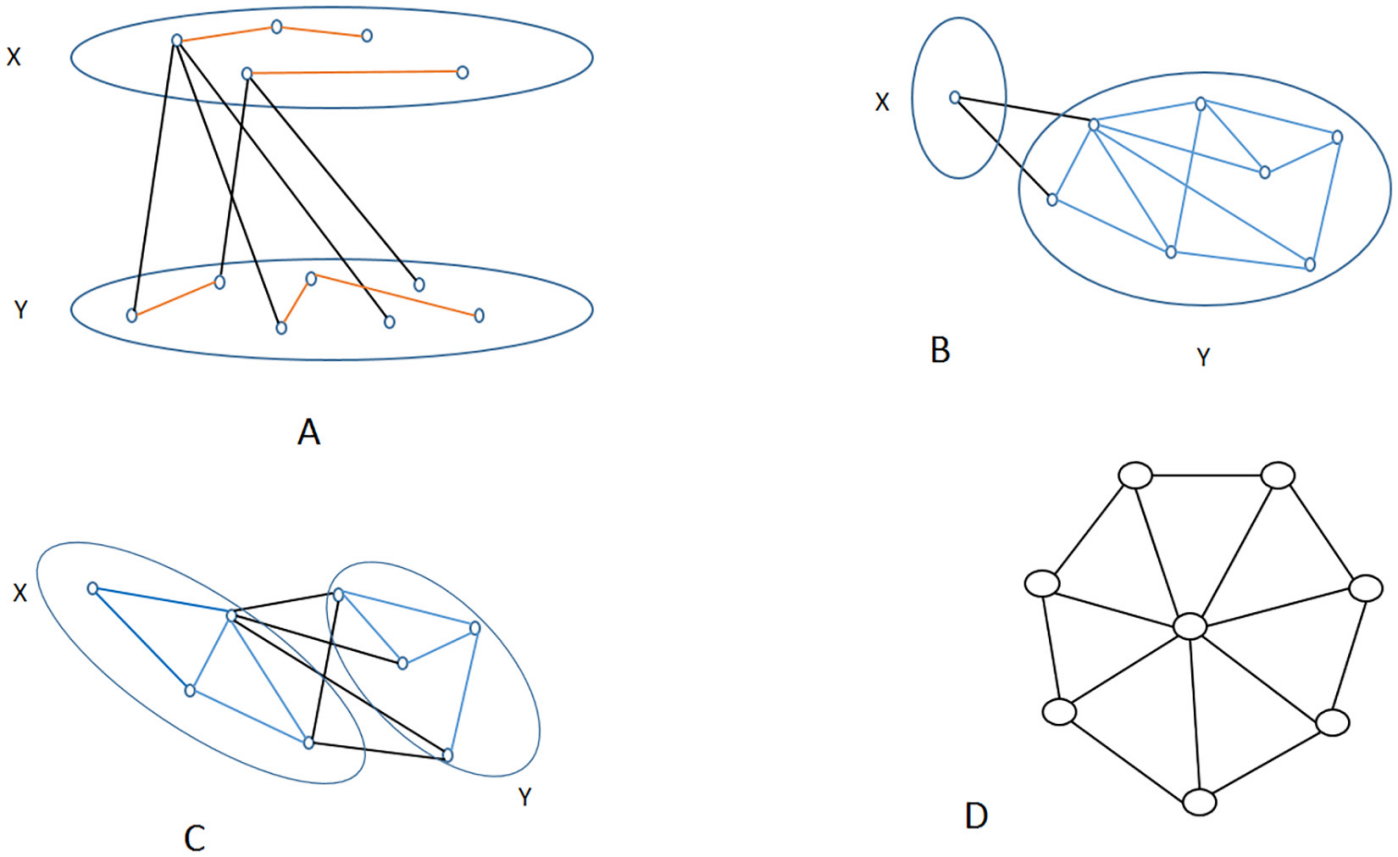
Panel A: An X-Y cut. The cut-edges are colored black. Panel B: An un-balanced minimum cut. Panel C: A balanced cut. Panel D: The wheel graph.

Suppose the nodes, or the vertices, of a graph are partitioned into two, disjoint, non-empty sets, say *X* and *Y*; their union is the whole vertex-set of the graph. The *X*, *Y* *cut* is the set of *all* edges connecting vertices of *X* with the vertices of *Y* (Fig 1 panel A). The size of the cut is the number of edges in the cut. In graph theory, the size of the minimum cut is an interesting quantity. The minimum cut between vertices *a* and *b* is the minimum cut, taken for all *X* and *Y*, where vertex *a* is in *X* and *b* is in *Y*. This quantity gives the “bottleneck”, in a sense, between those two nodes (c.f., Menger theorems and Ford-Fulkerson’s Min-Cut-Max-Flow theorem [20, 21]). The minimum cut in a graph is defined to be the cut with the fewest edges for *all* non-empty sets *X* and *Y*, partitioning the vertices.

Clearly, for non-negative weights, the size of the minimum cut in a non-connected graph is 0. Very frequently, however, in connected graphs, the minimum cut is determined by just the smallest degree node: that node is the only element of set *X* and all the other vertices of the graph are in *Y* (Fig 1 panel B). Because of this phenomenon, the minimum cut is frequently queried for the “balanced” case, when the size (i.e., the number of vertices) of *X* and *Y* needs to be equal (or, more exactly, may differ by at most one if the number of the vertices of the graph is odd), see Fig 1 panel C. This problem is referred to as *the balanced minimum cut* or the *minimum bisection* problem. If the minimum bisection is small that means that there exist a partition of the vertices into two sets of equal size that are connected with only a few edges. If the minimum bisection is large then the two half-sets in *every possible bisections* of the graph are connected by many edges.

Therefore, the balanced minimum cut of a graph is independent of the particular labeling of the nodes. The number of all the balanced cuts in a graph with *n* vertices is greater than

$$\frac{1}{n+1}2^{n},$$

that is, for *n* = 463, this number is much larger than the number of atoms in the visible universe [22]. Consequently, one cannot practically compute the minimum bisecton width by reviewing all the bisectons in a graph of that size. Moreover, the complexity of computing this quantity is known to be NP-hard [23] in general, but with contemporary integral programming approaches, and for the graph-sizes we are dealing with, the exact values are computable in reasonable time.

In computer engineering, an important measure of the quality of an interconnection network is its minimum bisection width [24]: the higher the width is the better the network. Based on this observation, we can say that the data imply the better quality of female connectome, compared to that of males.

For the whole brain graph, as it is anticipated, we have found that the minimum balanced cut is almost exactly represents the edges crossing the *corpus callosum*, connecting the two cerebral hemispheres.

We show that within both hemispheres, the minimum bisection size of female connectomes are significantly larger than the minimum bisection size of the males. Much more importantly, we show that this remains true if we *normalize with the sum of all edge-weights*: that is, *this phenomenon cannot be due* to the higher number of edges or the greater edge weights in the female brain: it is an intrinsic property of the female brain graph in our data analyzed.

For example, in the 234-vertex resolution, in the left hemisphere, the normalized balanced minimum cut in females, on the average, is 0.09416, in the males 0.07896, *p* = 0.00153 (see Table 1 with a summary and Tables 2, 3, 4, 5 and 6 with the results).

We think that this finding is one of the main results of the present work: even if the significant difference in the weighted edge numbers were due to some artifacts in the data acquisition/processing workflow, the normalized balanced minimum cut size seems to be independent from those processes.

### Eigengap and the expander property

Expander graphs and the expander-property of graphs are one of the most interesting area of graph theory: they are closely related to the convergence rate and the ergodicity of Markov chains, and have applications in the design of communication- and sorting networks and methods for de-randomizing algorithms [25]. A graph is an *ɛ*-expander, if every—not too small and not too large—vertex-set *S* of the graph has at least *ɛ*∣*S*∣ outgoing edges (see [25] for the exact definition).

Random walks on good expander graphs converge very fast to the limit distribution: this means that good expander graphs, in a certain sense, are “intrinsically better” connected than bad expanders. It is known that large eigengap of the walk transition matrix of the graph implies good expansion property [25].

We have found that women’s connectomes have significantly larger eigengap, and, consequently, they are better expander graphs than the connectomes of men. For example, in the 83-node resolution, in the left hemisphere and in the unweighted graph, the average female connectome’s eigengap is 0.306 while in the case of men it is 0.272, with *p* = 0.00458.

### The number of spanning forests

A *tree* in graph theory is a connected, cycle-free graph. Any tree on *n* vertices has the same number of edges: *n*−1. Trees, and tree-based structures are common in science: phylogenetic trees, hierarchical clusters, data-storage on hard-disks, or a computational model called *decision trees* all apply graph-theoretic trees. A *spanning tree* is a minimal subgraph of a connected graph that is still connected. Some graphs have no spanning trees at all: only connected graphs have spanning trees. A tree has only one spanning tree: itself. Any connected graph on *n* vertices has a minimum of *n*−1 and a maximum of *n*(*n*−1)/2 edges [26]. A connected graph with few edges still may have exponentially many different spanning trees: e.g., the *n*-vertex wheel on Fig 1 panel D has at least 2^*n*−1^ spanning trees (for *n* ≥ 4). Cayley’s famous theorem, and its celebrated proof with Prüfer codes [27] shows that the number of spanning trees of the complete graph on *n* vertices is *n* ^*n*−2^.

If a graph is not connected, then it contains more than one connected components. Each connected component has at least one spanning tree, and the whole graph has at least one *spanning forest*, comprises the spanning trees of the components. The number of spanning forests is clearly the product of the numbers of the spanning trees of the components.

For graphs in general, one can compute the number of their spanning forests by Kirchoff’s matrix tree theorem [28–31] using the eigenvalues of the Laplacian matrix [29] of the graph.

We show that female connectomes have significantly higher number of spanning trees than the connectomes of males. For example, in the 129-vertex resolution, in the left hemisphere, the logarithm of the number of the spanning forests in the unweighted case are 162.01 in females, 158.88 in males with *p* = 0.013.

**Fig 2:**
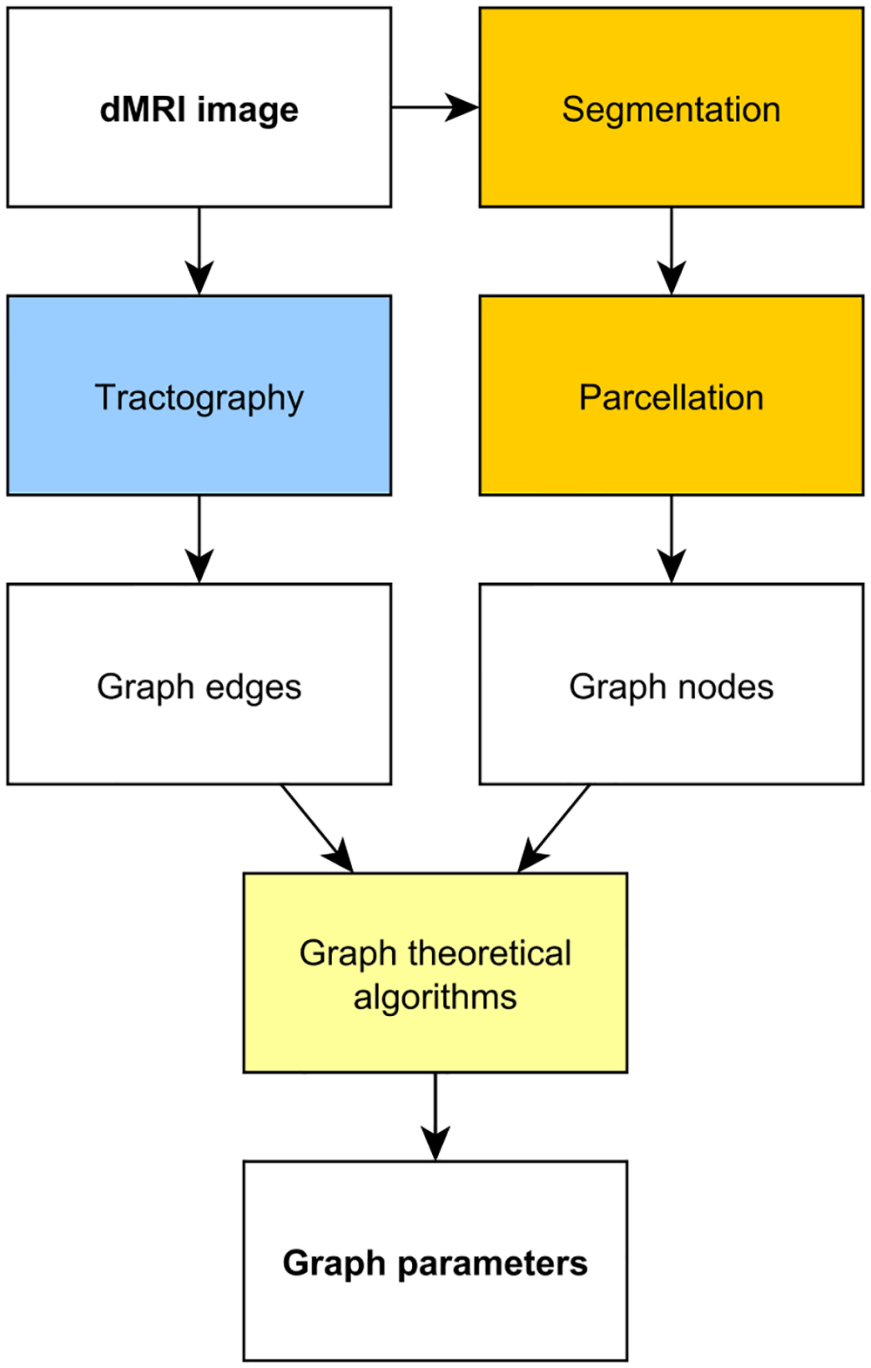
The block diagram of the workflow presented. The phases are detailed in the “Methods” section.

The workflow of this work is summarized on Fig 2.

**Fig 3:**
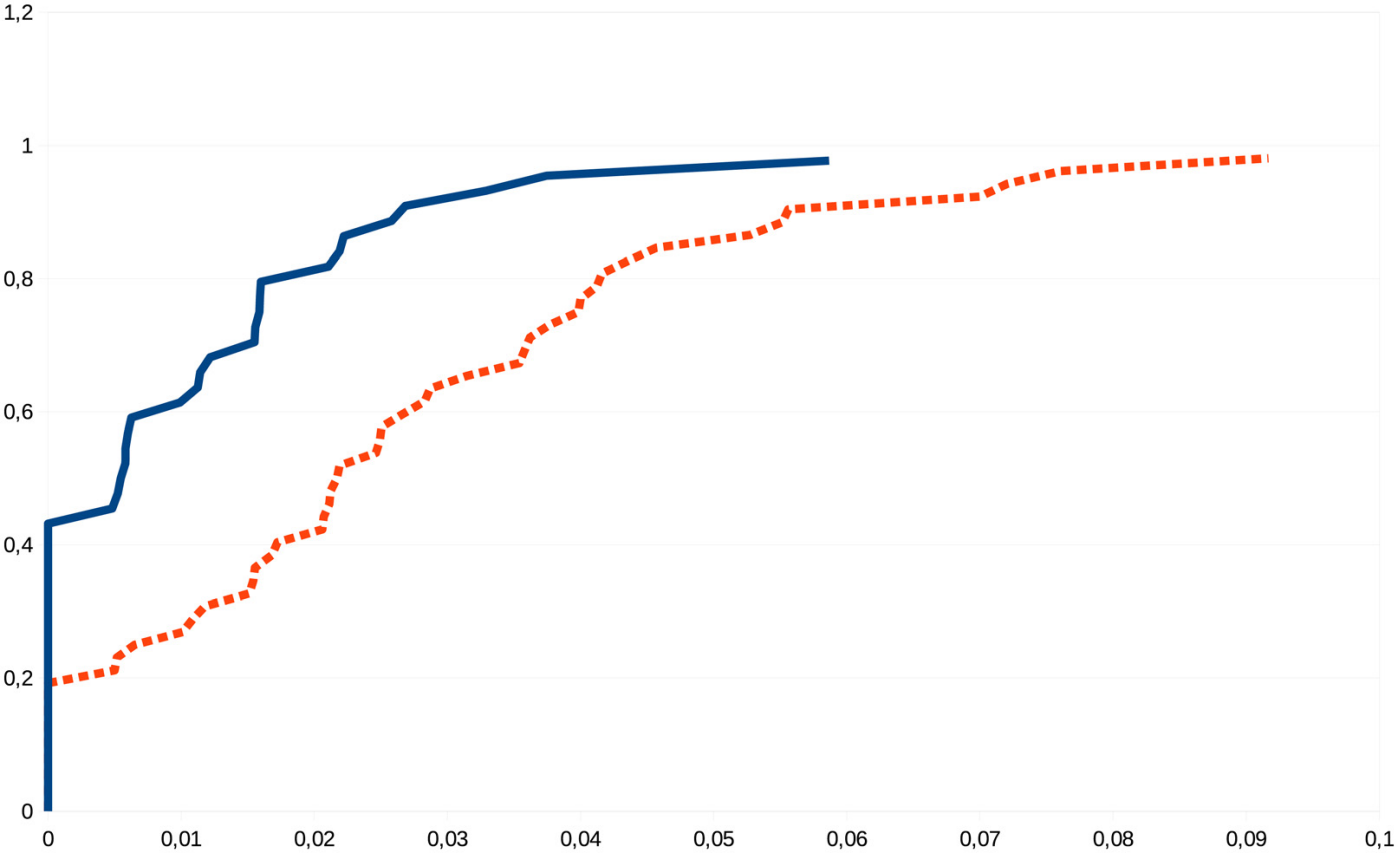
Empirical cumulative distribution function of the Right_MinCutBalDivSum_FAMean graph parameter (that is, edge-number-normed minimum bisection width in the right hemisphere, weighted by the arithmetic mean of the fractional anisotropies [35] of the fibers, belonging to the edge) in the 129-node resolution. For every value *x* on the horizontal line, the curves demonstrate the male (blue, continuous line) and female (red, dashed line) fraction of subjects with Right_MinCutBalDivSum_FAMean value of at most *x*. For example, for *x* = 0.02, 40% of the females have the Right_MinCutBalDivSum_FAMean value less than *x*, while about 85% of males have that value less than *x*.

**Fig 4:**
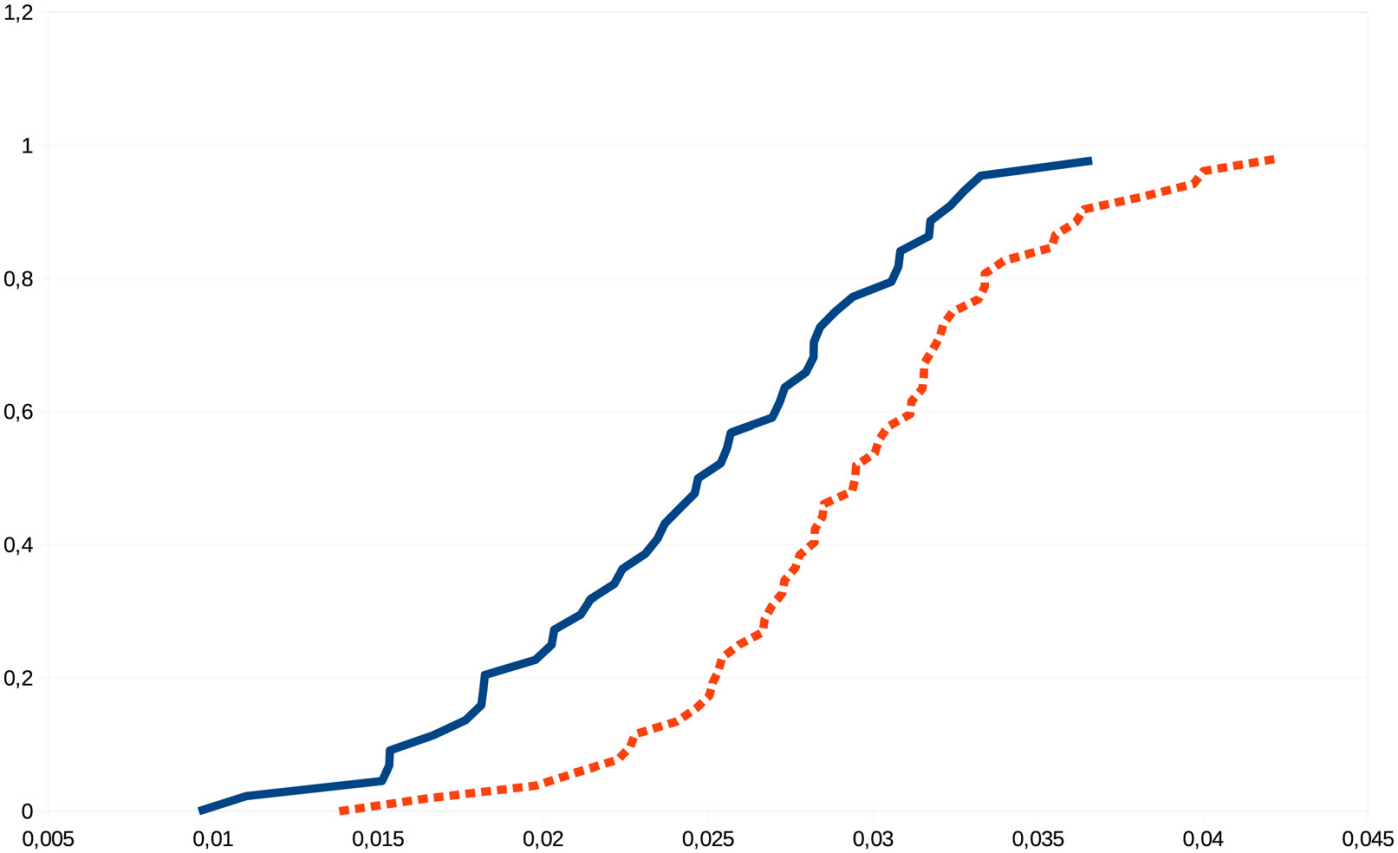
Empirical cumulative distribution function of the All_PGEigengap_FiberNDivLength graph parameter (that is, the eigengap of the transition-matrix of the whole brain graph with each edge weighted by the number of fibers belonging to the edge, divided by their average length), in the 129-node resolution. For every value *x* on the horizontal line, the curves demonstrate the male (blue, continuous line) and female (red, dashed line) fraction of subjects with All_PGEigengap_FiberNDivLength value of at most *x*. For example, for *x* = 0.025, about 17% of the females have the All_PGEigengap_FiberNDivLength value less than *x*, while about 58% of males have that value less than *x*.

Figs 3 and 4 visualize the differences of some graph parameters between the connectomes of the sexes.

## Methods

### Data source and graph computation

The dataset applied is a subset of the Human Connectome Project [32] anonymized 500 Subjects Release: (http://www.humanconnectome.org/documentation/S500) of healthy subjects between 22 and 35 years of age. Data was downloaded in October, 2014.

The Connectome Mapper Toolkit [33] (http://cmtk.org) was applied for brain tissue segmentation into grey and white matter, partitioning, tractography and the construction of the graphs from the fibers identified in the tractography step. The Connectome Mapper Toolkit [33] default partitioning was used (computed by the FreeSurfer, and based on the Desikan-Killiany anatomical atlas) into 83, 129, 234, 463 and 1015 cortical and sub-cortical structures (as the brainstem and deep-grey nuclei), referred to as “Regions of Interest”, ROIs, (see Fig 4 in [33]). Tractography was performed by the Connectome Mapper Toolkit [33], choosing the deterministic streamline method with the MRtrix processing tool [34] with randomized seeding.

The graphs were constructed as follows: the nodes correspond to the ROIs in the specific resolution. Two nodes were connected by an edge if there exists at least one fiber (determined by the tractography step) connecting the ROIs, corresponding to the nodes. More than one fibers, connecting the same nodes, may or may not give rise to the weight of that edge, depending on the weighting method. Loops were deleted from the graph.

The weights of the edges are assigned by several methods, taking into account the lengths and the multiplicities of the fibers, connecting the nodes:

- Unweighted: Each edge has weight 1.
- FiberN: The number of fibers traced along the edge: this number is larger than one if more than one fibers connect two cortical or sub-cortical areas, corresponding to the two endpoints of the edge.
- FAMean: The arithmetic mean of the fractional anisotropies [35] of the fibers, belonging to the edge.
- FiberLengthMean: The average length of the fibers, connecting the two endpoints of the edge.
- FiberNDivLength: The number of fibers belonging to the edge, divided by their average length. This quantity is related to the simple electrical model of the nerve fibers: by modeling the fibers as electrical resistors with resistances proportional to the average fiber length, this quantity is precisely the conductance between the two regions of interest. Additionally, FiberNDivLength can be observed as a reliability measure of the edge: longer fibers are less reliable than the shorter ones, due to possible error accumulation in the tractography algorithm that constructs the fibers from the anisotropy data. Multiple fibers connecting the same two ROIs, corresponding to the endpoints, add to the reliability of the edge, because of the independently tractographed connections.

By *generalized adjacency matrix* we mean a matrix of size *n* × *n* where *n* is the number of *nodes* (or *vertices*) in the graph, whose rows and columns correspond to the nodes, and whose each element is either zero if there is no edge between the two nodes, or equals to the weight of the edge connecting the two nodes. By the *generalized degree* of a node we mean the sum of the weights of the edges adjacent to that node. Note that the generalized degree of the node *v* is exactly the sum of the elements in the row (or column) of the generalized adjacency matrix corresponding to *v*. By *generalized Laplacian matrix* we mean the matrix *D*−*A*, where *D* is a diagonal matrix containing the generalized degrees, and *A* is the generalized adjacency matrix.

### Graph parameters

We calculated various graph parameters for each brain graph and weight function. These parameters included:

- Number of edges (Sum). The weighted version of this quantity is the sum of the weights of the edges.
- Normalized largest eigenvalue (AdjLMaxDivD): The largest eigenvalue of the generalized adjacency matrix, divided by the average degree. Dividing by the average degree of vertices was necessary because the largest eigenvalue is bounded by the average- and maximum degrees, and thus is considered by some a kind of “average degree” itself [26]. This means that a denser graph may have a bigger *λ*_*max*_ largest eigenvalue solely because of a larger average degree. We note that the average degree is already defined by the sum of weights.
- Eigengap of the transition matrix (PGEigengap): The transition matrix *P*_*G*_ is obtained by dividing all the rows of the generalized adjacency matrix by the generalized degree of the corresponding node. When performing a random walk on the graph, for nodes *i* and *j*, the corresponding matrix element describes the probability of transitioning to node *j*, supposing that we are at node *i*. The eigengap of a matrix is the difference of the largest and the second largest eigenvalue. It is characteristic to the expander properties of the graph: the larger the gap, the better expander is the graph (see [25] for the exact statements and proofs).
- Hoffman’s bound (HoffmanBound): The expression
  $$1+\frac{\lambda_{max}}{|\lambda_{min}|},$$
  where *λ*_*max*_ and *λ*_*min*_ denote the largest and smallest eigenvalues of the adjacency matrix. It is a lower bound for the chromatic number of the graph. The chromatic number is generally higher for denser graphs, as the addition of an edge may make a previously valid coloring invalid.
- Logarithm of number of spanning forests (LogAbsSpanningForestN): The number of the spanning trees in a connected graph can be calculated from the spectrum of its Laplacian [28, 29]. Denser graphs tend to have more spanning trees, as the addition of an edge introduces zero or more new spanning trees. If a graph is not connected, then the number of spanning forests is the product of the numbers of the spanning trees of the components. The parameter LogAbsSpanningForestN equals to the logarithm of the number of spanning forests in the unweighted case. In the case of other weight functions, if we define the weight of a tree by the product of the weights of its edges, then this parameter equals to the sum of the logarithms of the weights of the spanning trees in the forests.
- Balanced minimum cut, divided by the number of edges (MinCutBalDivSum): The task is to partition the graph into two sets whose size may differ from each other by at most 1, so that the number of edges crossing the cut is minimal. This is the “balanced minimum cut” problem, or sometimes called the “minimum bisection width” problem. For the whole brain graph, our expectation was that the minimum cut corresponds to the boundary of the two hemispheres, which was indeed proven when we analyzed the results.
- Minimum cost spanning tree (MinSpanningForest), calculated with Kruskal’s algorithm.
- Minimum weighted vertex cover (MinVertexCover): Each vertex should have a (possibly fractional) weight assigned such that, for each edge, the sum of the weights of its two endpoints is at least 1. This is the fractional relaxation of the NP-hard vertex-cover problem [36]. The minimum of the sum of all vertex-weights is computable by a linear programming approach.
- Minimum vertex cover (MinVertexCoverBinary): Same as above, but each weight must be 0 or 1. In other words, a minimum size set of vertices is selected such that each edge is covered by at least one of the selected vertices. This NP-hard graph-parameter is computed only for the unweighted case. The exact values are computed by an integer programming solver SCIP (http://scip.zib.de), [37, 38].

The 9 parameters above were computed for all five resolutions and for the left and the right hemispheres and also for the whole connectome, with all 5 weight functions (with the following exceptions: MinVertexCoverBinary was computed only for the unweighted case, and the MinSpanningTree was not computed for the unweighted case).

The results are detailed in an Excel table with 480 rows (5 rows of different resolutions for each brain) and 120 columns (7 parameters are computed for all 5 weight-functions for the left- and right hemispheres and the whole brain, one parameter for just one weight function, and one parameter for 4 weight functions only, that is 7 ⋅ 5 ⋅ 3+1 ⋅ 3+4 ⋅ 3 = 120) at the site http://uratim.com/bigtable1.zip

### Investigation for possible artifacts

We applied the very same graph construction method for all dMRI data sets, independently of the sex of the subjects. Surprisingly, we have found significant differences in numerous graph parameters between male and female brains, e.g., in the number of the edges in the connectome. We will review here a possible bias in the connectome construction, and we conclude that, by the best of our knowledge, it cannot cause the differences in the graph parameters.

One possible source of error could be the statistically different brain sizes of the sexes [18]. In the tractography step, when streamlines are progressed by the deterministic method from voxel to voxel, longer fibers may stop prematurely [39, 40]. Therefore, longer fibers may be harder to reconstruct. Since male brains are larger than the brain of the females, they contain longer fiber bundles that could be more difficult to reconstruct.

We have applied five different edge weighting methods. One of these is called FiberLengthMean that describes the average lengths of fibers that define the edge in question.

Clearly, the FiberLengthMean weight rewards the longer fibers and penalizes the shorter ones. Consequently, the advantage of the total sum of these weights of the edges in the case of women needs to be smaller or non-existing if the “premature stop” tractography bias were the cause of the edge number difference. The data below show that just the opposite holds true.

More exactly, let us consider Table 3, containing data with resolution 129:

All_Sum_FiberLengthMean female: 30670.09535 male: 28478.19852 p = 0.03582 f/m ratio: 1.07
All_Sum_Unweighted female: 1020.80769 male: 972.86667 p = 0.00026 f/m ratio: 1.049,

Here the unweighted ratio is smaller, meaning that weighting with the fiber lengths increases the advantage of the females!

Similarly, in Table 4, with resolution 234:

All_Sum_FiberLengthMean female: 51558.63408 male: 48397.55225 p = 0.05764, f/m ratio: 1,065
All_Sum_Unweighted female: 1826.03846 male: 1742.66667 p = 0.00063 f/m ratio: 1,048

Here, again, the unweighted ratio is smaller, meaning that weighting with the fiber lengths increases the advantage of the females. We believe that these figures make our results stronger, proving that females have longer and more connections in their connectome than males.

### Statistical analysis

Since each connectome was computed in multiple resolutions (in 83, 129, 234, 463 and 1015 nodes), we had five graphs for each brain. In addition, the parameters were calculated separately for the connectome within the left and right hemispheres as well, not only the whole graph, since we intended to examine whether statistically significant differences can be attributed to the left or right hemispheres. Each subjects’ brain was corresponded to 15 graphs (5 resolutions, each in the left and the right hemispheres, plus the whole cortex with sub-cortical areas) and for each graph we calculated 9 parameters, each (with the exceptions noted above) with 5 different edge weights. This means that we assigned 7 ⋅ 5 ⋅ 3+1 ⋅ 3+4 ⋅ 3 = 120 attributes to each resolution of the 96 brains, that is, 600 attributes to each brain.

The statistical null hypothesis [41] was that the graph parameters do not differ between the male and the female groups. As the first approach, we have used ANOVA (Analysis of variance) [42] to assign p-values for all parameters in each hemispheres and in each resolutions and in each weight-assignments.

Our very large number of attributes may lead to false negatives, i.e., to “type II” statistical errors: in other words, it may happen that an attribute, with a very small p-value may appear “at random”, simply because we tested a lot of attributes. In order to deal with “type II” statistical errors, we followed the route described below.

We divided the population randomly into two sets by the parity of the sum of the digits in their ID. The first set was used for making hypotheses and the second set for testing these hypotheses. This was necessary to avoid type II errors resulting from multiple testing correction. If we made hypotheses for all the numerical parameters, then the Holm-Bonferroni correction [43] we used would have unnecessarily increased the p-values. Thus we needed to filter the hypotheses first, and that is why we needed the first set. Testing on the first set allowed us to reduce the number of hypotheses and test only a few of them on the second set.

The hypotheses were filtered by performing ANOVA (Analysis of variance) [42] on the first set. Only those hypotheses were selected to qualify for the second round where the p-value was less than 1%. The selected hypotheses were then tested for the second set as well, and the resulting p-value corrected with the Holm-Bonferroni correction method [43] with a significance level of 5%.

In Table 1 those hypotheses rejected were highlighted in bold, meaning that *all* the corresponding graph parameters differ significantly in sex groups at a combined significance level of 5%.

We also highlighted (in italic) those p-values which were individually less than the threshold, meaning that these hypotheses can *individually* be rejected at a level of 5%, but it is very likely that *not all* of these graph parameters are significantly different between the sexes.

## Conclusions

We have computed 83-, 129-, 234-, 463- and 1015 vertex-graphs from the diffusion MRI images of the 96 subjects of 52 females and 44 males, between the age of 22 and 35. After a careful statistical analysis, we have found significant differences between certain graph parameters of the male and female brain graphs. Our findings show that the female brain graphs have generally more edges (counted with and without weights), have larger normalized minimum bisection widths in its hemispheres, are better expander graphs and have more spanning trees (counted with and without weights) than the connectomes of males (Table 1). We believe that in the future, due to the relatively small size of the underlying networks, graph theoretical methods could have a wide application spectrum in the analysis of the connectome.
